# Barriers and accessibility‐improving strategies in mental health services for persons with hearing or vision impairments: Perspectives from professionals and clients—A qualitative interview study

**DOI:** 10.1111/papt.70006

**Published:** 2025-08-13

**Authors:** Bastian Hardt, Thomas Heidenreich, Christina Hunger‐Schoppe, Nele Adam, Julia Heinen, Joana Hopf, Axel Röhlig, Johannes Michalak

**Affiliations:** ^1^ Department of Psychology and Psychotherapy Witten/Herdecke University Witten Germany; ^2^ Department of Social Work, Education, and Nursing Sciences University of Applied Sciences Esslingen Esslingen Germany

**Keywords:** accessibility, barriers, hearing impairment, mental health, participatory research, qualitative content analysis, vision impairment

## Abstract

**Objectives:**

Despite studies showing that persons with hearing impairments (HI) or vision impairments (VI) have an increased risk of developing mental health disorders, mental health services frequently are not accessible and suited to the specific needs of both populations. However, there is limited research addressing how mental health services can be improved to meet these needs.

**Design:**

This qualitative interview study explores barriers and strategies to improve accessibility in mental health services for persons with HI or VI in Germany, as viewed with a multi‐perspective approach.

**Methods:**

Using problem‐centred interviews, which combine a flexible interview guide with a focus on specific issues, we applied a participatory research approach to gather insights from 58 participants, including mental health professionals specialised in treating persons with HI or VI and persons with HI or VI with lived experience as clients in mental health services.

**Results:**

The qualitative content analysis yielded 44 inductive subcategories within a category system of seven deductive main categories. Key barriers included communication, limited visual accessibility and environmental challenges, while improvement strategies focused on tailored therapeutic adaptations as well as proactive and collaborative practices between therapists and clients.

**Conclusions:**

Our findings highlight the need for structural changes and expanded mental health services that cater to the specific needs of persons with sensory impairments. These insights should contribute to improving mental health services, training programs for professionals and emphasise the importance of including persons with HI or VI, both professionals and clients, in the participatory development of accessible care.

Globally, the prevalence of sensory impairments, such as hearing impairments (HI) and vision impairments (VI) is increasing. According to the World Health Organization (WHO, 2021), approximately 430 million people currently live with HI, a number projected to exceed 700 million by 2050. About 30% of persons with HI are over 60 years old. Additionally, the WHO (2019) reports that 2.2 billion people have some form of near or distance VI, with the majority being over 50 years old. Due to population growth and aging demographics, the risk of acquiring impairments is expected to rise in the future. Consequently, an increasing number of health care professionals (e.g. psychologists and psychotherapists) will or have already come into contact with persons with HI or VI.

Persons with HI or VI are considered vulnerable populations, most studies showing a similar increased risk of developing mental health issues or mental disorders compared to persons without disabilities (see Bailly et al., 2003; Brunes et al., 2019; Jiang et al., 2020; Shoham et al., 2019; Zheng et al., 2017). Studies have also reported lower quality of life and increased mental health issues (e.g. mental distress) in both HI and VI populations (Chia et al., 2004; Fellinger et al., 2005, 2012; Nordvik et al., 2018; Taylor et al., 2016; Williams et al., 2015). There is a clear need for specialised mental health services for both groups, and it is essential to compare the efficacy of these treatments with those designed for persons without disabilities. Research on mental health treatments for persons with HI or VI remains in its early stages (Bailly et al., 2003; Cosh et al., 2019; Hardt et al., 2024; Olusanya et al., 2014; van der Aa et al., 2016). Meta‐analyses indicate limited evidence for the effectiveness of psychological treatments (Hardt et al., 2024; van der Aa et al., 2016; Wang et al., 2022). Possible reasons for the low effects include moderate to low methodological quality of the studies, a lack of adaptation of treatments for both populations and insufficient focus on mental health and psychotherapeutic approaches.

The requirements and challenges concerning mental health services for both populations are viewed differently from various perspectives. At the *political level*, the United Nations' Convention on the Rights of Persons with Disabilities (CRPD, 2007) emphasises equal access to health services for persons with disabilities, including mental health care. It calls on states to ensure non‐discriminatory, informed and dignified treatment through adequate training and ethical standards for health professionals.

At the *level of mental h*ealth *services for persons with HI*, effective communication is a crucial, yet often challenging aspect. The preference for different forms of communication, whether spoken language, sign language or through interpreters, varies among persons with HI (Porter, 1999; Schmidt & Metzner, 2020; Zhang, McKeever, et al., 2013; Zhang, Zhou, et al., 2013). The literature states that mental health services and access to psychotherapy remain insufficient (Crowe, 2017; Leigh et al., 1996; Pollard, 1994; Pollard et al., 2014; Zhang, McKeever, et al., 2013; Zhang, Zhou, et al., 2013). Specifically, for deaf clients, communication challenges are exacerbated by the lack of sign language proficiency among health care providers (Porter, 1999; Schmidt & Metzner, 2020; Zhang, McKeever, et al., 2013; Zhang, Zhou, et al., 2013). Persons with HI often view deafness through the lens of their language and culture, thus preferring therapists who are knowledgeable in these areas (Gill & Fox, 2012; Langley, 2014; Schröder & Vereenhooghe, 2021; Steinberg et al., 1998).

Regarding *mental health services for persons with VI*, significant barriers include the legibility and accessibility of treatment materials (Braille, screen reader). Services are generally considered inadequate (Holloway et al., 2018), with structural barriers such as the lack of Braille menus in hospitals being particularly notable (Carlson et al., 2020). The accessibility of psychological treatments, including rejection of persons with VI, remains a significant issue (Reed et al., 2020; van der Ham et al., 2021). Fraser et al. (2019) emphasised the lack of social participation among persons with VI and the attitudes of psychotherapists towards this impairment (e.g. reluctance to engage and prejudice), noting the absence of adapted diagnostic procedures. They recommend that therapists reflect on their responses to clients with VI to ensure accessible treatment options, which necessitate specialists trained in these areas (Harvey, 2003; Olkin, 2007).

At the *level of disability communities*, organisations founded by persons with HI or VI underscore the importance of mental health services for both populations and advocate for the greater participation of persons who are affected themselves. The Belfast Statement on Mental Health and Deafness (2014) asserts the right to effective communication in mental health settings for persons with HI. The National Association of the Deaf (NAD, 2003) and the World Blind Union (WBU, 2022) advocate for more accessible services for persons with sensory impairments, providing guidelines for an accessible framework and the implementation of CRPD requirements for equal health care.

The current state of mental health services indicates a lack of specialised and adapted treatment for persons with HI or VI. In this context, we posted the questions of how current mental health care is provided and what best practice approaches by experienced therapists look like for both populations. In the present study, we aimed to gain a more in‐depth and multi‐perspective view on mental health services for persons with either HI or VI. To achieve these goals, we conducted a qualitative interview study utilising a participatory research approach that included both mental health professionals specialised in treating persons with HI or VI and persons with HI or VI who had lived experience as clients in mental health services. This approach facilitated comparisons of experienced barriers and strategies from different perspectives (i.e. HI and VI; professionals and clients). Within this framework, we addressed the following research questions to examine the practical experiences and expertise of our four study groups:

1. How do professionals and clients get access to mental health services for persons with HI or VI?
2. How do professionals and clients describe the accessibility of mental health services for persons with HI or VI, and what are the barriers?
3. What strategies do professionals and clients apply to improve accessibility in mental health services for persons with HI or VI?
4. How do professionals and clients reflect on their experiences in mental health services for persons with HI or VI, and what are suggestions for the future?

## METHOD

The current study got approval by the Ethics Committee of Witten/Herdecke University (Ethics Application No. S‐51/2021) and was conducted in accordance with national law and the tenets of the Declaration of Helsinki of 1975 (as revised). Informed consent was obtained from all participants.

### Positionality statement

Eight authors collaborated in this study; one with a VI. The first author (BH) would like to state: ‘I am legally blind myself. My visual impairment is congenital and I volunteer in a self‐help organization for persons with sensory impairments (PRO RETINA Deutschland). In my professional life as a psychologist, I have worked with both colleagues and participants with sensory impairments. It is important to me to raise awareness and promote mental health care for persons with sensory impairments. I acknowledge that my positionality influenced this study to some extent; my experiences as a person living with a disability proved to be important in conducting this interview study and including implications for improving participation’. To avoid positionality bias, the current study was conducted in a participatory approach including a collaborative research discourse between the eight authors and external experts (Busch et al., 2019). In questions related to persons with HI or VI, we consulted with experts with personal experience of HI or VI, who work in the field of sign language qualification or practice in specialised mental health services. All of the authors had some experience working with persons with disabilities. Several authors have experiences in conducting participatory research.

### Eligibility criteria

#### Professionals

The current study included mental health professionals such as licensed psychotherapists, psychological counsellors, and clinical psychologists or physicians who have special knowledge in the treatment or counselling of persons with HI or VI. Criteria for this group were being mentioned in publicly accessible lists of mental health services for persons with HI or VI (e.g. provided by self‐help associations) or a professional activity in an institution that offers specialised mental health services for persons with HI or VI (e.g. psychiatry and rehabilitation clinics). The treatment context could be either outpatient or inpatient.

#### Clients

The study included persons with documented HI or VI and current or past experience as a client in mental health services in one or more treatment settings (inpatient or outpatient). The grade of impairment was assessed according to the WHO criteria for HI and VI (Stevens et al., 2013; WHO, 2021; WHO, ICD‐10, 2010; WHO, ICD‐11, 2020). Participants included were all 18 years of age or older.

### Recruitment

Participants were searched and contacted via social media, mailing lists, public lists of therapists, self‐help organisations and specialised mental health institutions. Recruitment and implementation of the interviews were made as accessible as possible for all participants. For example, information material and informed consent forms have been offered to the participants in multiple accessible formats (e.g. word document, PDF, audio format and simpler language including images). Prior to their use, materials were reviewed to ensure participatory alignment. If it was not possible for participants to sign the consent form, we provided the opportunity to record the consent through an audio or video recording. We followed the sampling method of theoretical saturation to ensure a broad and diverse range of interviews across the four groups (Glaser & Strauss, 1967; Guest et al., 2006). Indicators for professionals included personal disability experience (without or with HI or VI), the variety of mental health approaches applied (e.g. behavioural, psychodynamic, psychoanalytic, systemic psychotherapy and counselling) and different treatment settings (outpatient and inpatient). For client interviews, indicators were the grade of impairment (ranging from hard of hearing to deafness or visual impairment to blindness), the types of mental health services used (e.g. behavioural, psychodynamic, psychoanalytic, systemic psychotherapy and counselling) and treatment settings (outpatient and inpatient). Our aim was to conduct at least 10–15 interviews for each group, given the small population sizes of both specialised professionals and persons with HI or VI, as well as the potential discomfort in discussing one's own therapy experiences during an interview.

### Participants

A total sample of 58 persons participated in this study. Interviews were conducted with four subsamples: (1) 19 professionals for persons with HI; (2) 12 professionals for persons with VI; (3) 14 clients with HI and (4) 13 clients with VI (Table S1).

The group of professionals for persons with HI included 17 licensed psychotherapists and 2 psychological counsellors. The treatment settings were inpatient settings, counselling centres for persons with HI and psychotherapy in outpatient settings. Four of the 19 professionals reported a personal HI, which ranged from severe to profound impairment. Professionals for persons with VI involved nine licensed psychotherapists, a psychological counsellor, a physician and a clinical psychologist. The setting of treatments was inpatient settings and psychotherapy in outpatient settings. Half of the professionals stated a personal VI, which ranged from blindness (visual acuity between .02 and light perception) to blindness (no light perception).

All clients reported their grade of impairment. Clients with HI: one person with moderate impairment; three persons with severe impairment; six persons with profound impairment and four persons with complete or total hearing loss/deafness. Clients with VI: four persons with moderate visual impairment; two persons with blindness with a visual acuity between .05 and .02; two persons with blindness with a visual acuity between .02 and light perception and five persons with blindness with no light perception. A congenital disorder was described by three clients with HI and by five clients with VI.

Every client reported at least one outpatient psychotherapy; multiple mental health treatment experiences were reported by 13 clients with HI and 8 clients with VI. Inpatient psychotherapy was reported by 10 clients with HI and 8 clients with VI.

### Interviews

This qualitative interview study was conceptualised, structured and conducted in order of problem‐centred interviews (Witzel, 1985, 2000) with a clear focus on barriers and strategies to improve accessibility in mental health services for persons with HI or VI and providing room for other related individual experiences. In addition, interviews for professionals contained elements of the problem‐centred expert interview of Döringer (2020) which is based on the problem‐centred interview by Witzel (1985, 2000) but also provides room for expert‐specific knowledge, individual impressions and experiences of the interviewees (i.e. psychotherapeutic approaches and adapted treatment contents). Interview guides for professionals as well as for clients were designed for the same research questions and had the same structure to enable an overall comparison of the results of both groups. Both interview guides are provided in their original German versions and in English translations (Appendix S1).

The interview guides were developed by the research team and discussed with external experts. It consisted of a short questionnaire, the main section of the interview, and a postscript. In this regard, our participatory approach involved discussing questions about content and accessible interview conduct with persons with HI or VI. The short questionnaire included questions about demographics, type of impairment and mental health service experiences as a professional or client. The main section of the interview consisted of four blocks which started with open initial questions on (1) providing/accessing mental health services for persons with HI or VI (e.g. professionals first treatment of persons with HI or VI or clients finding a psychotherapy); (2) accessibility and barriers in mental health services (e.g. personal experienced and generally expected barriers); (3) strategies to improve accessibility/overcome barriers (e.g. adapted treatment and individual resources) and (4) reflection on the experiences in mental health services for persons with HI or VI (e.g. personal learnings from the experience and suggestions for the future). For example, the initial question regarding accessibility was: ‘I would now like to talk with you about the topic of “accessibility” for persons with a hearing or vision impairment in psychotherapy’. I am especially interested in learning about your experiences on this topic. What does the term ‘accessibility’ mean to you in general? Please tell me about it and feel free to give examples’. At the end of the interview, participants could add additional information. The postscript includes information on the central content of the interview, situational and non‐verbal factors observed and anomalies and ideas for interpretation from the interviewer's perspective. The interview guides also included notes for interviewers to pause after a question or on how to address potential comprehension issues, including suggestions for alternative phrasing (e.g. difficulties in understanding the term accessibility).

### Data collection

All interviews were conducted in German or German sign language. To expand the range of potential participants and ensure accessibility and participation, interviews were conducted via phone, video meetings or the online chat function included in Zoom (Zoom Video Communications, Inc., 2021). Sign language and written interpreters were a part of conducting the interviews if they were needed. All participants could choose their preferred way of communication with the research group prior to the interview. The interpreters selected for this study had extensive prior experience supporting clients in mental health services. Before the study, a briefing was held with the first author to introduce the study aims and procedures. The interviews were conducted by the first author and four research assistants (BSc degree in Psychology). All interviewers were trained in qualitative research methods and the interview guides prior to conducting the interviews (e.g. literature, slides and practice via role‐playing). The five interviewers met regularly to discuss study progress and provide additional training and supervision.

The average duration of all 58 interviews was *M* = 91 min (*SD* = 32). We conducted 43 interviews in spoken language without interpreters, six with a written interpreter, one with a sign language interpreter and two via online chat function. Interviews involving interpreters or conducted via chat function typically required more time, included more pauses for all participants, and were often spread across two to three sessions. Interpreters received the interview guide in advance to familiarise themselves with the questions and clarify any uncertainties with the research team. After the interview, participants with client experience were informed about the possibility of talking to a psychotherapist if they found the interview stressful. All interviews were recorded and then transcribed according to established transcription rules (Kowal & O'Connell, 2004; Mergenthaler, 1992).

All data of the study such as files of participants, audio recordings and interview transcripts were stored at Witten/Herdecke University. The interviews were pseudonymised by codenames which the participants have chosen themselves. Identifying data of participants were removed from the transcripts. Study materials were locked via passwords accessible only for the research team. All interviews were fully transcribed and analysed using the same coding procedures, regardless of the mode of data collection (i.e. spoken language, interpreter‐mediated or conducted via chat function). The analysis focused on the content of participants' responses rather than the modality of communication. Written responses were coded in the same manner as spoken ones. Following each interview, all participants had the opportunity to review their pseudonymised transcripts and to make changes themselves, such as the deletion or addition of specific statements.

### Data analysis

The interviews were analysed via Qualitative Content Analysis (QCA) (Mayring, 2014, 2015, 2022) with a mixed procedure in combining deductive and inductive category assignment. We used MAXQDA 2022 for data analysis. Due to the study design of four subsamples, an overall category system of seven deductive main categories (MCs) was created to receive a common structure for the analysis (Table S2). These seven MCs were derived from our research questions and essential questions of the interview guide. Within this category structure, we performed an inductive approach that allowed for the emergence of innovative subcategories (SCs) to provide as much open space for possible results without limitations, and to emphasise the four groups' different perspectives (Table S3). Deductive and inductive categories were compared and discussed by the research team, employing investigator triangulation as a quality criterion (Mayring, 2015). All transcripts were double‐coded by at least two researchers. Coding discrepancies were resolved through in‐depth team discussions and consensus‐based decisions; a third coder was not required. In the results section, quotations were translated from German to English.

## RESULTS

Across all four groups, professionals and clients with HI or VI, the QCA identified a total of 44 inductive SCs within the seven deductive MCs. Figures S1–S6 provides a detailed visual representation of all MCs and SCs in relation to the study's four research questions. Each figure maps the deductive and inductive categories for one research question, including the number and titles of all SCs and their allocation to the corresponding MCs. These figures are intended to support the understanding of how the category system emerged during the analysis. The following results section is also structured according to the seven MCs.

### Providing/accessing mental health services for persons with HI or VI (MC1)

The initial question of the interview focused on accessing mental health care for persons with HI or VI from the perspective of professionals (providing care) or clients (seeking treatment) (Figure S1).

Professionals of both groups reported that their ‘personal disability’ (SC1) was a motivation to offer treatment for persons with HI or VI. Their ‘professional life’ (SC2) was also a point of first contact with treatment for both populations (e.g. in studies, internships, therapist training and engagements in self‐help organisations). Additional knowledge for treating persons with impairments was supported via ‘literature research’ (SC3), ‘exchange with persons with HI or VI’ (SC4) and ‘exchange with specialized professionals’ (SC5). Professionals for persons with HI also reported their ‘fascination of sign language’ (SC6) which started their interest to offer specialised treatment.

Both client groups mentioned ‘online search’ (SC7) (e.g. via lists of therapists), ‘recommendation from professionals’ (SC8) (e.g. social worker and primary care physician) and ‘word of mouth’ (SC9) (e.g. by other persons with disabilities) as starting points to get in contact with mental health services. Only clients with VI added the option ‘contact by phone’ (SC10). ‘Reasons for beginning therapy’ (SC11) ranged from personal reasons to coping with impairments in both groups.

### Accessibility (MC2)

All participants had the opportunity to state their ‘general understanding of accessibility’ (SC12) for opening the problem field of accessibility and barriers (Figure S2).

Professionals defined society‐wide accessibility through general access to information, mobility, education, social care, health care, absence of physical and communicative barriers and awareness of these barriers:

[…] equal access to the same offers and without any obstacles. So that I can manage and carry it out on my own and don't necessarily have to be taken by the hand. (professional VI)

Clients added aspects such as inclusion, equal rights, personal independence and participation in daily life. ‘Doubts about accessibility’ (SC13) were mentioned by professionals and clients in the context of VI, they described accessibility as ‘utopia’ or ‘illusion’.

### Accessibility of mental health services (MC3)

All groups stated ‘specifications of accessibility in mental health services’ (SC14). Professionals specified the importance of unrestricted access to information and treatment, assistance with filling in documents, appropriate forms of communication for persons with HI and reachability of therapists for persons with VI:

Being able to access information and access therapy in the same way as someone without a disability. (professional HI)

Clients emphasised the role of selectable forms of communication and to independently complete therapy materials.

### Barriers (MC4)

Initially, barriers for persons with HI or VI in mental health services were categorised into two main categories: personally experienced barriers and generally expected barriers to capture a broader range of expertise. However, the coding process revealed that most barriers were mentioned in both main categories; leading to a content summary being made into a single main category (MC4) (Figure S3). For improved clarity, the following section presents the 13 SCs of MC4 organised into four thematic clusters: structural and systemic, accessibility and material‐related, therapy‐related and interpersonal barriers.

#### Structural and systemic barriers

This cluster comprises three subcategories: ‘finding or contacting a therapist’ (SC15), ‘availability of specialized services’ (SC16) and ‘waiting time’ (SC17).

Already prior to the beginning of treatment, barriers were present in ‘finding or contacting a therapist’ (SC15). Professionals specifically stated limited contact options for persons with HI (e.g. therapists are only reachable by phone) and noted difficulties in accessing information about therapy options (e.g. where to find lists of specialised therapists), persons with VI face challenges in accessing information (e.g. visually inaccessible websites and difficulties in navigating lists visually). Clients with HI emphasised contact by phone as a barrier when no other option was available. Clients with VI added difficulties in both outpatient and inpatient registration situations (e.g. not being allowed to enter with a guide dog or the staff being overwhelmed by interacting with persons with VI).

Professionals highlighted ‘availability of specialized services’ (SC16), particularly in relation to the lack of therapy in diverse communication forms (e.g. sign language), and the therapists' lack of experience in the field of treating persons with VI. Clients also mentioned a shortage of specialised services and the expected lack of disability‐related topics in therapist training.

‘Waiting time’ (SC17) was reported by professionals to generally be longer for both populations and to be further increased by limited treatment options. Clients waited up to several years to access general treatment and identified their limited mobility as a contributing factor.

#### Accessibility and material‐related barriers

This cluster includes four subcategories: ‘route to therapy’ (SC18), ‘building conditions’ (SC19), ‘visual access in treatment’ (SC25) and ‘assistive devices’ (SC26).

All groups reported ‘route to therapy’ (SC18) as an expected barrier. Professionals noted long distances to reach treatment and clients with VI added the challenges of transportation (e.g. being unable to drive and use unknown routes of public transportation).

‘Building conditions’ (SC19) posed challenges for persons with HI or VI when navigating facilities or perceiving their therapist. In the context of VI, professionals and clients highlighted orientation and mobility barriers in inpatient settings (e.g. lack of Braille markings and inadequate lighting). Clients with HI reported poor room acoustics and lighting that affected communication (e.g. spoken language, lip reading and sign language).

‘Visual access in treatment’ (SC25) was only detected for persons with VI. For example, professionals described general difficulties in seeing therapeutic materials and time lost due to reading content aloud, whereas clients reported difficulties in not seeing the therapist face and mimic.

‘Assistive devices’ (SC26) encountered problems in treatment situations. Professionals for persons with VI stated issues due to limited battery life and functionality of magnifiers and screen readers (e.g. not always suitable for scaling or reading materials with tables and graphs). Clients with HI mentioned malfunctions, battery depletion and noise originating from their hearing devices (e.g. hearing aids or microphone systems), whereas clients with VI added the general missing of assistive devices at mental health services.

#### Therapy‐related barriers

This cluster contains three subcategories: ‘knowledge of disability culture’ (SC20), ‘therapy content and procedures’ (SC21) and ‘challenges and limitations’ (SC27).

‘Knowledge of disability culture’ (SC20) was identified as a barrier related to therapists. Professionals suspected barriers in the fact that therapists know little about the life experiences, conditions, socialisation and culture of persons with HI or VI (e.g. sign language, deaf culture or how a blind person navigates daily life). Clients with VI mentioned that therapists had misconceptions or incorrect knowledge about persons with disabilities (e.g. either underestimating or overestimating client's capabilities).

‘Therapy content and procedures’ (SC21) revealed barriers within different treatment methods and was noted similarly by professionals and clients. Treatment for persons with HI was limited in cases where interventions relied on visual components, such as Eye Movement Desensitization and Reprocessing (EMDR), which was problematic as these clients needed to see the therapist's mouth for lip reading. Additionally, clients with HI encountered difficulties with completing questionnaires or worksheets due to challenges with written language comprehension. Similarly, clients with VI described reading and completing treatment materials as barriers, alongside limitations in participating specific treatment options, particularly in inpatient settings (e.g. occupational therapy, sports therapy and gestalt therapy):

Worksheets that we can't read, that we can't fill out, and some anamnesis forms that we can't complete. (client VI)

All four groups experienced personal ‘challenges and limitations’ (SC27). Professionals mentioned limitations in treatment of clients with severe communicative and multiple impairments or struggled to reconcile their role as therapists with a disability themselves. Clients reported frustration, isolation and overall exclusion from therapeutic content:

It was definitely a limitation for me not to be able to fully participate in all the exercises or to experience them in the way I would have liked. (client VI)

#### Interpersonal barriers

This cluster includes three subcategories: ‘interpersonal relationship’ (SC22), ‘third person in therapy’ (SC23) and ‘communication’ (SC24).

Professionals and clients identified ‘interpersonal relationship’ (SC22) as a barrier within treatment situations, which included aspects such as reluctance to engage and prejudices regarding persons with disabilities. Professionals reported misjudgments regarding the educational level of clients (e.g. based on emails from clients who primarily use sign language), as well as emotional reactions (e.g. activating a fear of developing a HI or VI in the future):

The biggest difficulties are on the emotional level, such as prejudices, insecurities, and also defensive reactions from colleagues who have no experience with this. (professional VI)

These experiences were also reported by clients. All groups mentioned issues regarding a lack of trust in the therapist and the therapist's non‐belonging to the disability community from the clients' perspective (e.g. therapists without a disability are perceived as unable to understand blindness or hearing therapists are seen as trying to exert power over a deaf patient due to the dominance of hearing individuals in society).

‘Third person in therapy’ (SC23) such as interpreters (e.g. sign language interpreters and written interpreters) were reported as barriers by professionals for persons with HI due to a lack of privacy or misinterpreting. Clients with HI rejected the support of interpreters because of their privacy. Clients with VI reported having to rely on family members as companions to attend therapy, which led to personal conflicts and issues of privacy.

‘Communication’ (SC24) was the most specific barrier identified for persons with HI. Professionals emphasised longer duration of therapy due to difficulties in communication, explaining therapeutic concepts and procedures to clients with limited sign language proficiency or when clients relied solely on lip reading. All clients with HI experienced communication barriers and highlighted the mental effort required to process spoken language or sign language during therapy, and challenges with therapists who spoke very quietly or unclearly:

Hearing is mental work, and it's just tiring to listen to someone who speaks very softly, someone you constantly have to remind that you have difficulty hearing. (client HI)

Clients with VI reported barriers in nonverbal communication (e.g. facial strain or effort to visually focus was misinterpreted as an angry expression, leading to conflicts in therapy).

To address the challenges described above, professionals and clients developed a variety of strategies to overcome these barriers. The following sections outline these approaches, offering practical insights into improving the accessibility of mental health services for persons with HI or VI (Figures S4 and S5).

### Professionals' strategies to improve accessibility (MC5)

Professionals for persons with HI mentioned the use of ‘visualization techniques’ (SC28) to explain concepts during therapy or they utilised tangible objects, such as coffee cups, to help patients answer questions related to 'theory of mind' abilities (e.g. complemented by constellation work, drawing objects or using flipcharts and video materials).

‘Shared communication strategies’ (SC29) were applied by professionals for persons with HI to establish a common language within therapy. Professionals and clients with HI described adapting the speed of spoken language or sign language, clearer articulation and establishing communication rules. Especially for deaf clients, professionals mentioned strategies such as explaining terms used in psychotherapy or finding alternative terms that were better understood, and, when necessary, adjusting the language level to address comprehension difficulties:

Sometimes we even make something up ourselves. If we know what we want to say and they don't know a sign for it, then we invent one. (professional HI)

‘Multiple contact options’ (SC30) were introduced to address the difficulties of contacting therapists by phone, offering written language and video options for persons with HI (e.g. email, messaging platforms or video calls for lipreading or sign language).

Professionals emphasised ‘awareness of HI culture’ (SC31) as essential knowledge for therapists to better understand the identity development and socialisation of persons with HI (i.e. identification as a hard of hearing or deaf person, more oriented towards spoken language or sign language and belonging to the hearing society or deaf culture):

When did a person become deaf? Did they identify as hearing or deaf? A lot of psychological distress depends on this, and it can also be reduced if the issue of identity is addressed. (professional HI)

‘Environmental modifications and support’ (SC32) were noted by all four groups. Building conditions of mental health services must be accessible for corresponding clients. Especially in inpatient settings, it was emphasised that in addition to orientation aids such as guiding systems, good acoustics and lighting, the provision of assistive devices is beneficial (e.g. FM sound‐systems for better hearing in group therapy). The staff should also be adequately prepared to support clients with HI or VI in their orientation (e.g. therapists, caregivers or reception personnel).

‘Route descriptions and transportation’ (SC33) was stated by professionals and clients in context of VI. Professionals provided detailed and target group‐adapted directions (e.g. via website or upon request), and clients additionally reported that their health insurance covered the cost of taxi rides to therapy.

‘Adaptation of therapy materials’ (SC34) and ‘adjustments within therapy’ (SC35) were reported for treating both groups. Professionals adapted their materials for persons with HI, particularly those who are sign‐language‐oriented (e.g. by reducing text, using images or simpler language). Professionals working with persons with VI modified the format of documents (e.g. digital materials and large print or text in Braille). Clients with VI mentioned the willingness of therapists to complete documents together with them. Within therapy, professionals for persons with HI highlighted the increased explanation of psychotherapeutic content (e.g. through psychoeducation) or the adaptation of methods (e.g. conducting relaxation exercises with open eyes), whereas professionals for persons with VI utilised more auditory or tactile methods and fewer written materials (e.g. reading content aloud and creating a tactile mood barometer). Both client groups highlighted benefits of online therapy sessions (e.g. no need for transportation and improved perception through the use of assistive devices at home), and the therapist's explanation of cues and therapy content (e.g. verbal or non‐verbal reactions in individual and group therapy).

Both professional groups reported ‘professional development’ (SC36), emphasising ongoing exchange and networking with experts in the field of working with persons with HI or VI (e.g. continuing education, supervision, intervision, conferences and self‐help organisations).

‘Resources and strengths of professionals’ (SC37) were mentioned by all four groups. Characteristics, such as patience, conscientiousness, empathy, openness and a sensitive approach to the topic of disability, the therapists' own background or experiences with disabilities, were highlighted.

#### Summary

- In working with clients with HI, mental health professionals use visualisations, collaboratively establish shared forms of communication, and integrate knowledge related to Deaf and disability culture into therapy.
- In working with clients with VI, mental health professionals adapt therapy to focus on auditory content, ensure access to materials and address orientation and mobility needs.
- To promote inclusive care for both groups, professionals and institutions emphasise barrier‐free environments and use disability‐specific training and support.

### Clients' strategies to improve accessibility (MC6)

‘Proactive engagement’ (SC38) was identified as one of the key strategies for clients of both groups to actively participate in treatment and address identified barriers in therapy. Professionals emphasised their practice of asking how best to communicate or structure information for clients. However, the majority of participants reported that it was often the clients themselves who took the initiative:

When I had difficulties, I directly addressed my therapist, saying, 'Hey, this isn't quite right.' It was never a problem. I was also encouraged to bring up any issues directly if there was something. (client HI)

I told the psychotherapist that we should sit the way I preferred, and I also addressed his facial expressions, and with that, the issue was resolved. (client VI)

In contrast to third‐party involvement in therapy, various systems of ‘personal support’ (SC39) were highlighted by all groups. Professional support was provided through communication via sign language interpreters, written interpreters or staff members who assisted with filling out documents (e.g. registration forms, questionnaires and worksheets). Individuals from the clients' personal life were perceived as supportive (e.g. driving clients to therapy, handling phone calls and accompanying them as support in the initial sessions).

‘Peer contact’ (SC40) was mentioned by professionals for persons with VI, who recommended self‐help groups and self‐help organisations as a complement to therapy (e.g. exchange about overcoming barriers).

All groups described ‘using personal devices’ (SC41) as a helpful measure that enhances auditory or visual access to therapy (e.g. microphone systems for hearing aids, notebooks or smartphones with Braille displays and screen readers):

‘If I hadn't had the technology that I personally use, I wouldn't have been able to do the therapy.’ (client HI)

All groups reported ‘resources and strengths of clients’ (SC42) as supporting factors in therapy. Clients demonstrated motivation for change, directness in communication, perseverance, resilience and the ability to handle setbacks and develop or adapt their own coping strategies.

#### Summary

- Both clients with HI or VI proactively engage in the therapeutic process to address disability‐related topics and collaboratively work with their therapist towards more accessible treatment.
- Clients benefit from disability‐related support in mental health services—both professional and personal—as well as from integrating assistive technologies and additional peer contact.

### Overall reflection (MC7)

At the end of the interviews, all groups reflected on ‘personal learnings’ (SC43) in mental health services for persons with HI or VI (Figure S6).

Professionals reported that they had grown by overcoming challenges in therapy and that engaging with disability culture had enriched their experiences and knowledge. Although clients reported having encountered both positive and negative aspects of mental health services, in both groups, it was noted that the current system, however, is not designed for persons with disabilities and included experiences of rejection:

Before I became deaf, I wasn't aware that psychotherapy isn't accessible to everyone […] now I know the full extent, and it's very distressing because it's tied to so much suffering. The system is simply not designed to remove additional barriers and to address individual needs. (client HI)

‘Suggestions for improvement’ (SC44) underscored current barriers and how to overcome them in the future. All four groups emphasised the need for more specialised services and professionals for persons with HI or VI within both outpatient and inpatient settings. In addition to these suggestions, political and systemic changes were advocated (e.g. incorporating sign language in standard schools, treatment of persons with disabilities as a topic in training programs for therapists and more research in this field).

## DISCUSSION

This study revealed various barriers and strategies to improve accessibility from the perspectives of professionals and clients related to mental health services for persons with HI or VI. First, results of the interviews will be discussed in light of our research questions, previous research and limitations of this study, including implications for future research. Second, we will present implications for mental health practice and draw conclusions from our findings.

Professionals demonstrated a commitment to specialised treatment for persons with HI or VI, motivated by personal disability experiences or initial contacts made through their professional journeys. Clients discovered therapy options through online research; although personal interactions were often essential. Access to treatment is particularly challenging for persons with HI, primarily due to the requirement for initial telephone contact (Schröder & Vereenhooghe, 2021). Persons with VI reported experiences of rejection that further complicated their access to therapy, such as negative experiences with health care providers that made participants feel misunderstood or not taken seriously (van der Ham et al. (2021).

General mental health services are perceived to be not sufficiently accessible for both populations. Communication barriers were the most significant issue for professionals and clients in the context of HI, which is in line with the literature (Schröder & Vereenhooghe, 2021). Communication barriers often stem from non‐specialised therapy offerings missing multiple communication forms or support options preferred by clients with HI (e.g. sign language or interpreters) (Porter, 1999; Schmidt & Metzner, 2020; Zhang, McKeever, et al., 2013; Zhang, Zhou, et al., 2013). Previous findings that highlighted cultural mistrust and communication breakdowns between deaf clients and hearing therapists were also emphasised in our reports from professionals and clients (Gill & Fox, 2012; Langley, 2014; Schröder & Vereenhooghe, 2021; Steinberg et al., 1998).

For clients with VI, barriers are primarily interpersonal and arise from technical, content‐related or structural barriers. The comprehensive reports from participants regarding content‐related barriers underscore the need for accessible therapy materials and specific interventions (Zhang, McKeever, et al., 2013, Zhang, Zhou, et al., 2013). Especially in inpatient settings, our results underlined subjective evidence for structural barriers that the accessibility of treatment such as psychotherapy is still an issue (Carlson et al., 2020; Reed et al., 2020; van der Ham et al., 2021). Our findings regarding interpersonal barriers are particularly noteworthy. This theme from our interviews corresponds to results from Fraser et al. (2019) focus group study that concluded that persons with VI often face stereotyping, leading to exclusion and rejection.

In addition, third persons in therapy were viewed with ambivalence in this study, as the confidential nature of psychotherapy suggests it should be conducted independently. Clients with HI reported a lack of privacy during therapy sessions supported by interpreters. Though clients with VI more often rely on support persons for transportation, reading or filling out documents, especially the role of support persons in their therapy contexts remains underexplored.

Professionals in our study emphasised culturally sensitive approaches, shared communication and visualisation techniques as key strategies in treating persons with HI (Chapman & Dammeyer, 2017; Davidson et al., 2012; Landsberger & Diaz, 2011). In this process, professionals and clients go beyond the mere use of different forms of communication (e.g. spoken language, sign language and communicating through interpreters). They actively seek and establish a shared language that not only enhances communication within therapy but, in some cases, makes it possible in the first place (e.g. adapting speech rate and volume and finding new signs for therapeutic content). Therapeutic limitations for clients with HI could potentially be mitigated through video‐based therapy options. In this respect, Crowe (2017) suggests that clients are generally receptive to telehealth offerings. In our study, these insights are also applicable to clients with VI.

In the field of VI, key strategies to improve eyesight, legibility and accessibility to mental health services were still strongly emphasised in our study, as already mentioned for inpatient treatment settings (Carlson et al., 2020; Holloway et al., 2018). The independent participation of clients with VI played a highly important role (e.g. filling out questionnaires alone without relying on others). Professionals and treatment facilities provided low‐barrier conditions to improve medical standards for persons with VI, in line with the requirements of the CRPD (2007). Furthermore, professional development and training are viewed as urgently necessary, emphasised by both professional groups (Harvey, 2003; Olkin, 2007).

One key strategy which provided new insights from our study was that both client groups contribute to overcoming barriers by proactively expressing individual problems and disability‐related needs in treatment to improve inclusive care. Professionals similarly aid in this process through openness towards disability‐centered topics, actively questioning and improving the accessibility of their treatment. Through interaction and a trusting therapeutic relationship, barriers can be collaboratively managed.

In reflecting on their personal experiences, both clients with HI or VI may hold more critical perceptions of mental health services compared to professionals. Professionals trained specifically to work with these populations, however, express particularly negative views towards non‐specialised services and broader systemic barriers within mental health care. Both clients and professionals reported positive experiences when therapy is tailored to individual needs. All groups consequently expressed a desire for expanded and specialised therapeutic offerings in the future. The findings indicate that persons with HI or VI make significant efforts to overcome barriers; however, they frequently encounter structural and interpersonal challenges, particularly in inpatient and non‐specialised contexts.

### Limitations

The study showed its strength in multiple perspectives and in‐depth insights on mental health services for persons with HI or VI but also included several limitations. The predominantly retrospective interviews may lead to biases, such as memory errors, potentially affecting the credibility of the recalled therapy experiences (e.g. selectively recalled perceived barriers). Results could be skewed by socially desirable responses (e.g. professionals emphasised their therapeutic expertise or inclusive approaches in an overly positive way), although measures were taken to mitigate this through carefully conducted interviews (e.g. open‐ended questions, encouraging reflection and avoiding leading questions). It is also important to note that the experiences described were derived from the German health care system. Although it could be expected that comparable challenges and barriers are present in health care systems of other nations, future studies should carefully investigate the similarities and differences of the psychological treatment of clients with HI or VI in different countries.

The results indicate that the subcategories of professionals and clients in the field of VI show higher consistency compared to those in the field of HI. Professionals for clients with HI more frequently reported their sign language proficiency and their therapeutic experience with deaf clients, whereas only one sign‐language‐oriented person was among the interviewed clients and the client group predominantly reported therapy experiences conducted in spoken language. This imbalance may have influenced which barriers and strategies were emphasised, particularly underrepresenting clients who primarily use sign language. Furthermore, a generally low number of male participants was included in the study, possibly due to the higher prevalence of women seeking psychotherapy (Kuehner, 2017) and the predominance of female mental health professionals in the field (El‐Gabalawy & Katz, 2013), which may limit the generalisability of gender‐related insights.

Moreover, it should be noted that the results of our study do not apply to persons with deafblindness (e.g. Usher syndrome). Dual sensory impairment is a distinct and heterogeneous disability population that, in our opinion, requires its own research.

Future research should build upon this study and further explore the perspectives of each of the four subgroups individually to identify additional barriers and accessibility strategies. Particular attention should be given to sign‐language‐oriented clients and therapists working with interpreters or support persons, as their specific needs and relational dynamics remain underexplored. Targeted recruitment strategies will also be needed to include more male participants and to access underserved populations such as deafblind individuals (e.g. through self‐advocacy and support organisations). In addition, future research should include non‐specialised professionals to identify systemic barriers and support the development of more inclusive care for persons with HI or VI. Longitudinal or observational methods may offer deeper insight into the development of inclusive therapeutic processes over time.

### Implications

The findings of this study yield practical recommendations for mental health facilities as well as for the training and education of therapists and clinic staff.

As highlighted in our results, structural barriers within therapeutic settings must be dismantled. Greater consideration should be given to guidelines of self‐help organisations from both communities. For instance, the World Federation of the Deaf (2020) and the World Blind Union (2022) have already formulated measures for accessible conditions in therapeutic settings and culturally sensitive communication practices (e.g. guidance systems within buildings, accessible documents and sign‐language‐oriented therapists).

In line with the political requirements of the CRPD (2007), incorporating courses on psychotherapy for clients with HI and VI into psychotherapeutic training can help to improve skills and reduce discomfort. Engaging with defensive reactions regarding the topic of disability within self‐reflection would also be beneficial. Current specialised therapists could also act as role models, mentors and instructors. For example, in the field of professional development, the Qualified Mental Health American Sign Language (ASL) Interpreter certification program of the University of Kentucky: This program equips professionals with both mental health expertise and proficiency in ASL, enabling them to provide accessible communication and culturally sensitive care during therapy sessions. To meet the need for adequately trained professionals, we recommend the integration of disability‐related knowledge already at the academic level. In subsequent psychotherapy training programs, disability should be addressed as a mandatory topic. In addition, active mental health professionals should have access to structured professional development opportunities—ideally digital and in‐person, and with training formats tailored to specific types of impairments.

For therapeutic work in mental health services with persons with HI or VI, it is particularly important to be aware that professionals should adapt their treatment to the individual needs and challenges of the clients, and that this process greatly benefits from continuous communication and active involvement of the clients, which is highly appreciated (Glickman, 2008; Olkin, 2017). Establishing easily accessible and comprehensive lists of specialised therapists for persons with HI or VI should be offered by mental health organisations. Accessibility guidelines would ensure a consistent approach to inclusion and development (Hardt et al., 2024).

#### Key practical recommendations

- Availability of specialised, accessible and disability‐sensitive mental health services for persons with HI or VI, aligned with established standards and community‐specific recommendations.
- Inclusion of disability‐related content in academic curricula, psychotherapy training and continuing education with formats tailored to specific types of impairments.
- Participatory and inclusive mental health care involves clients actively shaping therapy and includes professionals and persons with lived experience in the development of services, in various roles (e.g. as role models, experts, advisors or researchers).

## CONCLUSIONS

This qualitative interview study, which incorporated multiple perspectives and a participatory research approach, uncovered numerous barriers as well as strategies for overcoming them. We hope that our findings contribute valuable expert knowledge to the current state of research and inspire mental health providers to pursue further education in the treatment of persons with HI or VI. Our results indicate that addressing barriers often begins within the therapeutic context; but we also emphasise the need for broader structural and personnel changes. Expanding and continuously improving mental health services specifically for persons with sensory impairments should be a primary objective moving forward.

## AUTHOR CONTRIBUTIONS

**Bastian Hardt:** Conceptualization; writing – original draft; writing – review and editing; methodology; formal analysis; investigation; project administration; data curation. **Thomas Heidenreich:** Methodology; writing – review and editing; formal analysis; writing – original draft. **Christina Hunger‐Schoppe:** Conceptualization; writing – review and editing; methodology. **Nele Adam:** Investigation; formal analysis; software. **Julia Heinen:** Investigation; software; formal analysis. **Joana Hopf:** Investigation; software; formal analysis. **Axel Röhlig:** Investigation; software; formal analysis. **Johannes Michalak:** Conceptualization; writing – original draft; writing – review and editing; methodology.

## CONFLICT OF INTEREST STATEMENT

The authors report no conflicts of interest, financial disclosures or funding received for this work. The authors alone are responsible for the content and writing of the article.

## ETHICS STATEMENT

The current study got approval by the Ethics Committee of Witten/Herdecke University (Ethics Application No. S‐51/2021), and was conducted in accordance with national law and the tenets of the Declaration of Helsinki of 1975 (as revised). Informed consent was obtained from all participants (in written or audio form).

## Supporting information


Figure S1

Table S1

Table S2

Table S3

Appendix S1

## Data Availability

Due to the sensitive nature of the data collected in this study, which involves personal and confidential information from therapists and patients, the datasets (including interview transcripts) are not publicly available. Access to the raw data is restricted to protect participant privacy in accordance with ethical guidelines and consent agreements.

## References

[papt70006-bib-0002] Bailly, D. , Dechoulydelenclave, M. B. , & Lauwerier, L. (2003). Hearing impairment and psychopathological disorders in children and adolescents. Review of the recent literature. Encephale, 29(4), 329–337. 14615703

[papt70006-bib-0003] Belfast Statement on Mental Health and Deafness . (2014). 6th World Congress on Mental Health and Deafness . https://wcmhd2014.esmhd.org/

[papt70006-bib-0004] Brunes, A. , Hansen, M. B. , & Heir, T. (2019). Post‐traumatic stress reactions among individuals with visual impairments: A systematic review. Disability and Rehabilitation, 41(18), 2111–2118. 10.1080/09638288.2018.145988 29644887

[papt70006-bib-0005] Busch, M. D. , Jean‐Baptiste, E. , Person, P. F. , & Vaughn, L. M. (2019). Activating social change together: A qualitative synthesis of collaborative change research, evaluation and design literature. Gateways: International Journal of Community Research and Engagement, 12(2), 1–26.

[papt70006-bib-0006] Carlson, C. , Howe, T. , Pedersen, C. , & Yoder, L. H. (2020). Caring for visually impaired patients in the hospital: A multidisciplinary quality improvement project. The American Journal of Nursing, 120(5), 48–55. 10.1097/01.naj.0000662820.87519.52 32332367

[papt70006-bib-0007] Chapman, M. , & Dammeyer, J. (2017). The significance of deaf identity for psychological well‐being. Journal of Deaf Studies and Deaf Education, 22(2), 187–194. 10.1093/deafed/enw073 27881482

[papt70006-bib-0008] Chia, E. M. , Wang, J. J. , Rochtchina, E. , Smith, W. , Cumming, R. R. , & Mitchell, P. (2004). Impact of bilateral visual impairment on health‐related quality of life: The Blue Mountains Eye Study. Investigative Ophthalmology & Visual Science, 45(1), 71–76. 10.1167/iovs.03-0661 14691156

[papt70006-bib-0009] Cosh, S. , Helmer, C. , Delcourt, C. , Robins, T. G. , & Tully, P. J. (2019). Depression in elderly patients with hearing loss: Current perspectives. Clinical Interventions in Aging, 14, 1471–1480. 10.2147/CIA.S195824 31616138 PMC6698612

[papt70006-bib-0010] Crowe, T. V. (2017). Is telemental health services a viable alternative to traditional psychotherapy for deaf individuals? Community Mental Health Journal, 53(2), 154–162. 10.1007/s10597-016-0025-3 27260308

[papt70006-bib-0011] Davidson, F. , Cave, M. , Reedman, R. , Briffa, D. , & Dark, F. (2012). Dialectical behavioral therapy informed treatment with deaf mental health consumers: An Australian pilot program. Australasian Psychiatry: Bulletin of Royal Australian and New Zealand College of Psychiatrists, 20(5), 425–428. 10.1177/1039856212458981 23014125

[papt70006-bib-0012] Döringer, S. (2020). The problem‐centered expert interview. Combining qualitative interviewing approaches for investigating implicit expert knowledge. International Journal of Social Research Methodology, 24(3), 265–278. 10.1080/13645579.2020.1766777

[papt70006-bib-0013] El‐Gabalawy, R. , & Katz, J. (2013). Gender differences in the experience of mental health professionals and their clients. International Journal of Mental Health Systems, 7(1), 25. 10.1186/1752-4458-7-25 24188964 PMC4174904

[papt70006-bib-0014] Fellinger, J. , Holzinger, D. , Dobner, U. , Gerich, J. , Lehner, R. , Lenz, G. , & Goldberg, D. (2005). Mental distress and quality of life in a deaf population. Social Psychiatry and Psychiatric Epidemiology, 40(9), 737–742. 10.1007/s00127-005-0936-8 16143834

[papt70006-bib-0015] Fellinger, J. , Holzinger, D. , & Pollard, R. (2012). Mental health of deaf persons. Lancet, 379(9820), 1037–1044. 10.1016/S0140-6736(11)61143-4 22423884

[papt70006-bib-0016] Fraser, S. , Beeman, F. S. , & Southall, K. (2019). Stereotyping as a barrier to the social participation of older adults with low vision: A qualitative focus group study. BMJ Open, 9, e029940. 10.1136/bmjopen-2019-029940 PMC6731881 31481561

[papt70006-bib-0017] Gill, I. J. , & Fox, J. R. (2012). A qualitative meta‐synthesis on the experience of psychotherapy for deaf and hard‐of‐hearing people. Mental Health, Religion and Culture, 15(6), 637–651. 10.1080/13674676.2011.609161

[papt70006-bib-0018] Glaser, B. G. , & Strauss, A. L. (1967). The discovery of grounded theory: Strategies for qualitative research. Aldine Publishing Company. 10.1093/sf/46.4.555

[papt70006-bib-0019] Glickman, N. (2008). Cognitive‐behavioral therapy for deaf and hearing persons with language and learning challenges. Taylor & Francis.

[papt70006-bib-0020] Guest, G. , Bunce, A. , & Johnson, L. (2006). How many interviews are enough? An experiment with data saturation and variability. Field Methods, 18(1), 59–82. 10.1177/1525822X05279903

[papt70006-bib-0021] Hardt, B. , Graser, J. , Heidenreich, T. , & Michalak, J. (2024). A systematic review and meta‐analysis of psychological interventions for persons with hearing or vision impairment: Research gaps and call to action. Clinical Psychology: Science and Practice, 32(2), 135–154. 10.1037/cps0000229

[papt70006-bib-0022] Harvey, M. A. (2003). Psychotherapy with deaf and hard of hearing persons: A systemic model (2nd ed.). Routledge. 10.4324/9781410607386

[papt70006-bib-0023] Holloway, E. , Sturrock, B. , Lamoureux, E. , Keeffe, J. , Hegel, M. , Casten, R. , Mellor, D. , & Rees, D. (2018). Can we address depression in vision rehabilitation settings? Professionals' perspectives on the barriers to integrating problem‐solving treatment. Disability and Rehabilitation, 40(3), 287–295. 10.1080/09638288.2016.1250172 27868437

[papt70006-bib-0024] Jiang, F. , Kubwimana, C. , Eaton, J. , Kuper, H. , & Bright, T. (2020). The relationship between mental health conditions and hearing loss in low‐ and middle‐income countries. Tropical Medicine & International Health, 25(6), 646–659. 10.1111/tmi.13393 32219942

[papt70006-bib-0025] Kowal, S. , & O'Connell, D. C. (2004). Zur Transkription von Gesprächen. In U. Flick , E. von Kardorff , & I. Steinke (Eds.), Qualitative Forschung: Ein Handbuch (pp. 437–447). Rowohlt.

[papt70006-bib-0026] Kuehner, C. (2017). Why do women suffer from depression more than men? The role of gender‐related factors. The Lancet Psychiatry, 4(2), 146–158. 10.1016/S2215-0366(16)30338-1 27856392

[papt70006-bib-0027] Landsberger, S. A. , & Diaz, D. R. (2011). Identifying and assessing psychosis in deaf psychiatric patients. Current Psychiatry Reports, 13(3), 198–202. 10.1007/s11920-011-0186-2 21327903

[papt70006-bib-0127] Langley, J. F. (2014). Hearing the deaf: An exploration of mental health services for deaf adults from a three‐party perspective. Alliant International University ProQuest Dissertations Publishing (3624144).

[papt70006-bib-0028] Leigh, I. W. , Corbett, C. A. , Gutman, V. , & Morere, D. A. (1996). Providing psychological services to deaf individuals: A response to new perceptions of diversity. Professional Psychology: Research and Practice, 27(4), 364–371. 10.1037/0735-7028.27.4.364

[papt70006-bib-0030] Mayring, P. (2014). Qualitative content analysis: Theoretical foundation, basic procedures and software solution. Klagenfurt. https://nbn‐resolving.org/urn:nbn:de:0168‐ssoar‐395173

[papt70006-bib-0031] Mayring, P. (2015). Qualitative Inhaltsanalyse: Grundlagen und Techniken (2nd ed.). Beltz.

[papt70006-bib-0032] Mayring, P. (2022). Qualitative Inhaltsanalyse. Grundlagen und Techniken (13. überarbeitete Auflage). Beltz.

[papt70006-bib-0033] Mergenthaler, E. (1992). Die Transkription von Gesprächen. Eine Zusammenstellung von Regeln mit einem Beispielskript . Ulmer Textbank. Universität Ulm.

[papt70006-bib-0034] National Association of the Deaf . (2003). Position statement on mental health services . https://www.nad.org/about‐us/position‐statements/position‐statement‐on‐mental‐health‐services/

[papt70006-bib-0035] Nordvik, Ø. , Laugen Heggdal, P. O. , Brännström, J. , Vassbotn, F. , Aarstad, A. K. , & Aarstad, H. J. (2018). Generic quality of life in persons with hearing loss: A systematic literature review. BMC Ear, Nose and Throat Disorders, 18, 1. 10.1186/s12901-018-0051-6 29386982 PMC5778781

[papt70006-bib-0036] Olkin, R. (2007). Disability‐affirmative therapy and case formulation: A template for understanding disability in a clinical context. Counseling and Human Development, 39(8), 1–21.

[papt70006-bib-0037] Olkin, R. (2017). Disability‐affirmative therapy: A case formulation template for clients with disabilities. Oxford University Press.

[papt70006-bib-0038] Olusanya, B. O. , Neumann, K. J. , & Saunders, J. E. (2014). The global burden of disabling hearing impairment: A call to action. Bulletin of the World Health Organization, 92(5), 367–373. 10.2471/BLT.13.128728 24839326 PMC4007124

[papt70006-bib-0039] Pollard, R. Q. (1994). Public mental health service and diagnostic trends regarding individuals who are deaf or hard of hearing. The Special Issue on Deafness, 38(4), 147–160. 10.1037/h0080318

[papt70006-bib-0040] Pollard, R. Q. , Sutter, E. , & Cerulli, C. (2014). Intimate partner violence reported by two samples of deaf adults via a computerized American Sign Language survey. Journal of Interpersonal Violence, 29(5), 948–965. 10.1177/0886260513505703 24142445 PMC4161008

[papt70006-bib-0041] Porter, A. (1999). Sign‐language interpretation in psychotherapy with deaf patients. American Journal of Psychotherapy, 53(2), 163–176. 10.1176/appi.psychotherapy.1999.53.2.163 10415986

[papt70006-bib-0042] Reed, N. S. , Assi, L. , Pedersen, E. , Alshabasy, Y. , Deemer, A. , Deal, J. A. , Willink, A. , & Swenor, B. K. (2020). Accompaniment to healthcare visits: The impact of sensory impairment. BMC Health Services Research, 20(1), 990. 10.1186/s12913-020-05829-8 33121483 PMC7594990

[papt70006-bib-0043] Schmidt, S. , & Metzner, F. (2020). Psychotherapie für taube Patienten durch hörende Psychotherapeuten mittels Gebärdensprachdolmetscher – Ein Systematisches Literaturreview zu Forschungsbefunden und Empfehlungen [Psychotherapy for deaf patients by hearing psychotherapists using sign language interpreters – A systematic review on research findings and recommendations]. Gesundheitswesen, 82(2), 180–187. 10.1055/a-1033-7449 31810109

[papt70006-bib-0044] Schröder, L. , & Vereenhooghe, L. (2021). Mixed‐methods‐Studie zu Barrieren in der ambulanten Psychotherapie von Gehörlosen [Mixed‐methods study on barriers in outpatient psychotherapy for the deaf]. Psychotherapeut, 66, 62–69. 10.1007/s00278-020-00476-0

[papt70006-bib-0045] Shoham, N. , Lewis, G. , Favarato, G. , & Cooper, C. (2019). Prevalence of anxiety disorders and symptoms in people with hearing impairment: A systematic review. Social Psychiatry and Psychiatric Epidemiology, 54, 649–660. 10.1007/s00127-018-1638-3 30547211

[papt70006-bib-0046] Steinberg, A. G. , Sullivan, V. J. , & Loew, R. C. (1998). Cultural and linguistic barriers to mental health service access: The deaf consumer's perspective. The American Journal of Psychiatry, 155(7), 982–984. 10.1176/ajp.155.7.982 9659872

[papt70006-bib-0047] Stevens, G. , Flaxman, S. , Brunskill, E. , Mascarenhas, M. , Mathers, C. D. , Finucane, M. , & Global Burden of Disease Hearing Loss Expert Group . (2013). Global and regional hearing impairment prevalence: An analysis of 42 studies in 29 countries. European Journal of Public Health, 23(1), 146–152. 10.1093/eurpub/ckr176 22197756

[papt70006-bib-0048] Taylor, D. J. , Hobby, A. E. , Binns, A. M. , & Crabb, D. P. (2016). How does age‐related macular degeneration affect real‐world visual ability and quality of life? A systematic review. BMJ Open, 6(12), e011504. 10.1136/bmjopen-2016-011504 PMC5168634 27913556

[papt70006-bib-0049] UN General Assembly . (2007). *Convention on the Rights of Persons with Disabilities*: *resolution/adopted by the General Assembly* , 24 January 2007, A/RES/61/106.

[papt70006-bib-0050] van der Aa, H. P. , Margrain, T. H. , van Rens, G. H. , Heymans, M. W. , & van Nispen, R. M. (2016). Psychosocial interventions to improve mental health in adults with vision impairment: Systematic review and meta‐analysis. Ophthalmic & Physiological Optics: The Journal of the British College of Ophthalmic Opticians (Optometrists), 36(5), 584–606. 10.1111/opo.12313 27580757

[papt70006-bib-0051] van der Ham, A. J. , van der Aa, H. P. , Verstraten, P. , van Rens, G. H. M. B. , & van Nispen, R. M. A. (2021). Experiences with traumatic events, consequences and care among people with visual impairment and post‐traumatic stress disorder: A qualitative study from The Netherlands. BMJ Open, 11(2), 1–9. 10.1136/bmjopen-2020-041469 PMC7925917 33542039

[papt70006-bib-0052] Wang, B. , Gould, R. L. , Kumar, P. , Pikett, L. , Thompson, B. , Gonzalez, S. C. , & Bamiou, D. E. (2022). A systematic review and meta‐analysis exploring effects of third‐wave psychological therapies on hearing‐related distress, depression, anxiety, and quality of life in people with audiological problems. American Journal of Audiology, 31(2), 487–512. 10.1044/2022_AJA-21-00162 35549513

[papt70006-bib-0053] Williams, K. C. , Falkum, E. , & Martinsen, E. W. (2015). A cognitive therapy program for hearing‐impaired employees suffering from mental distress. International Journal of Audiology, 54(4), 227–233. 10.3109/14992027.2014.958621 25328031 PMC4438347

[papt70006-bib-0054] Witzel, A. (1985). Das problemzentrierte Interview (pp. 227–255). Beltz.

[papt70006-bib-0055] Witzel, A. (2000). Das problemzentrierte Interview. Forum Qualitative Sozialforschung, 1(1), 22. 10.17169/fqs-1.1.1132

[papt70006-bib-0057] World Blind Union . (2022). Accessibility = Go! A guide to action . https://worldblindunion.org/programs/accessibility/accessibility‐go‐a‐guide‐to‐action/

[papt70006-bib-0058] World Federation of the Deaf . (2020). Human rights of the deaf . https://wfdeaf.org/human‐rights/

[papt70006-bib-0059] World Health Organization . (2010). H54 visual impairment including blindness (binocular or monocular). In International statistical classification of diseases and related health problems (10th ed.). ICD‐10‐GM. https://icd.who.int/browse10/2010/en#/H54.0

[papt70006-bib-0060] World Health Organization . (2019). World report on vision . [8 October 2019].

[papt70006-bib-0061] World Health Organization . (2020). 9D90 vision impairment including blindness. In International statistical classification of diseases and related health problems (11th ed.). ICD‐11. https://icd.who.int/browse11/l‐m/en

[papt70006-bib-0062] World Health Organization . (2021). World report on hearing . [3 March 2021].

[papt70006-bib-0063] Zhang, M. , Zhou, H. , Zhang, J. , Guo, Y. , Wang, X. , & Wang, N. (2013). Evaluating the effects of hearing aids combined with psychological counseling on tinnitus in patients with deafness. Journal of Clinical Otorhinolaryngology, 27(10), 461–464. 23937007

[papt70006-bib-0064] Zhang, X. , McKeever, K. B. , Cotch, M. F. , & Roy, M. (2013). Association between depression and functional vision loss in persons 20 years of age or older in the United States, NHANES 2005–2008. JAMA Ophthalmology, 131(5), 573–581. 10.1001/jamaophthalmol.2013.2597 23471505 PMC3772677

[papt70006-bib-0065] Zheng, Y. , Wu, X. , Lin, X. , & Lin, H. (2017). The prevalence of depression and depressive symptoms among eye disease patients: A systematic review and meta‐analysis. Scientific Reports, 7, 46453. 10.1038/srep46453 28401923 PMC5388862

[papt70006-bib-0066] Zoom Video Communications, Inc . (2021). zoom. Verfügbar unter. https://zoom.us/.

