# Supplementary material for: Barriers and accessibility‐improving strategies in mental health services for persons with hearing or vision impairments: Perspectives from professionals and clients—A qualitative interview study
Source: Psychol Psychother. 2025 Aug 13;99(1):40–59. doi: 10.1111/papt.70006 (PMC12905524; doi:10.1111/papt.70006)
Supplement: Supplementary file 1 — Figure S1 [file PAPT-99-40-s002.pdf]

Figure 1

Main category MC1 including subcategories SC1 to SC11

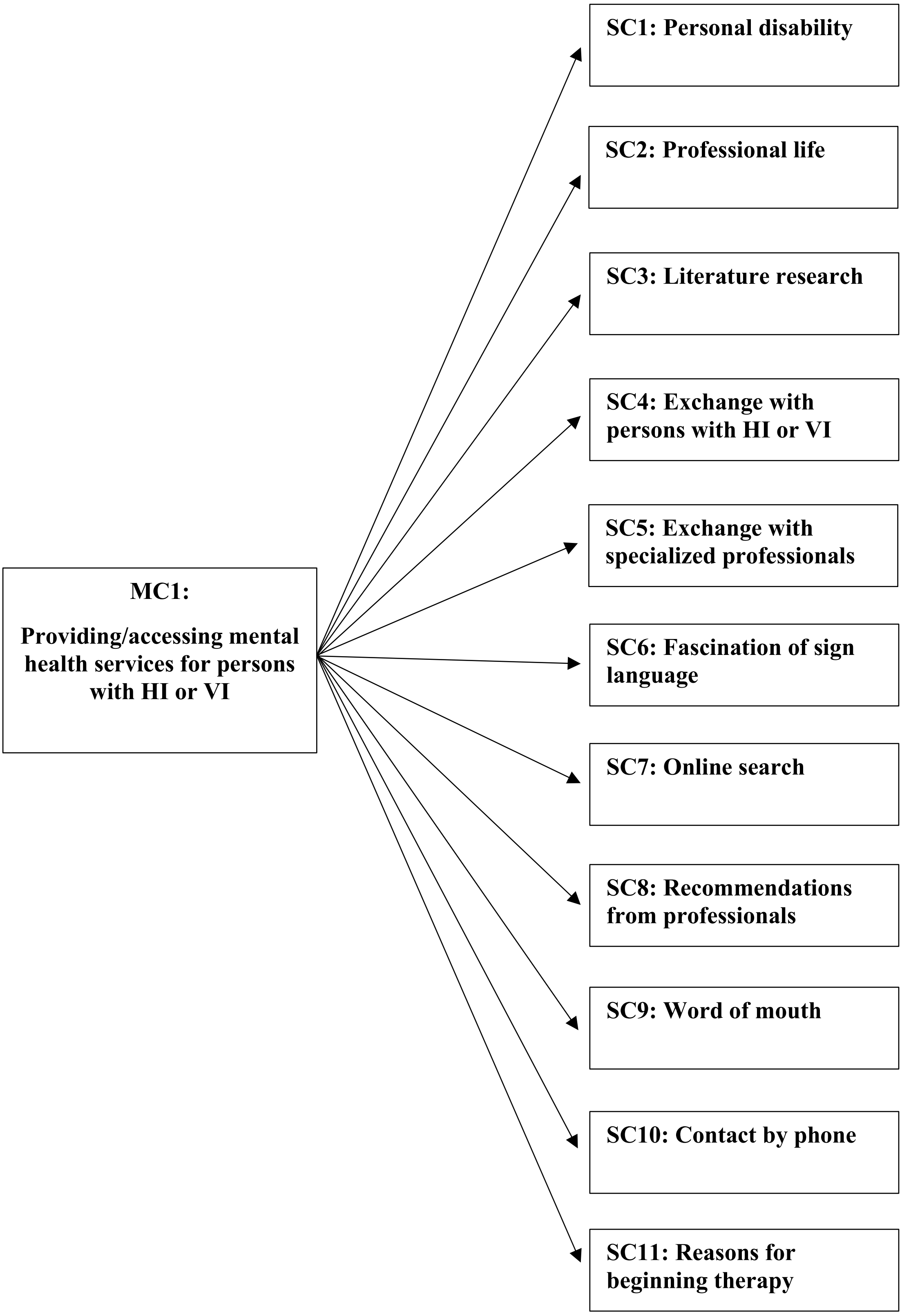

Note: Research question 1: How do professionals and clients get access to mental health services for persons with HI or VI?

Figure 2

Main categories MC2 and MC3 including subcategories SC12 to SC14

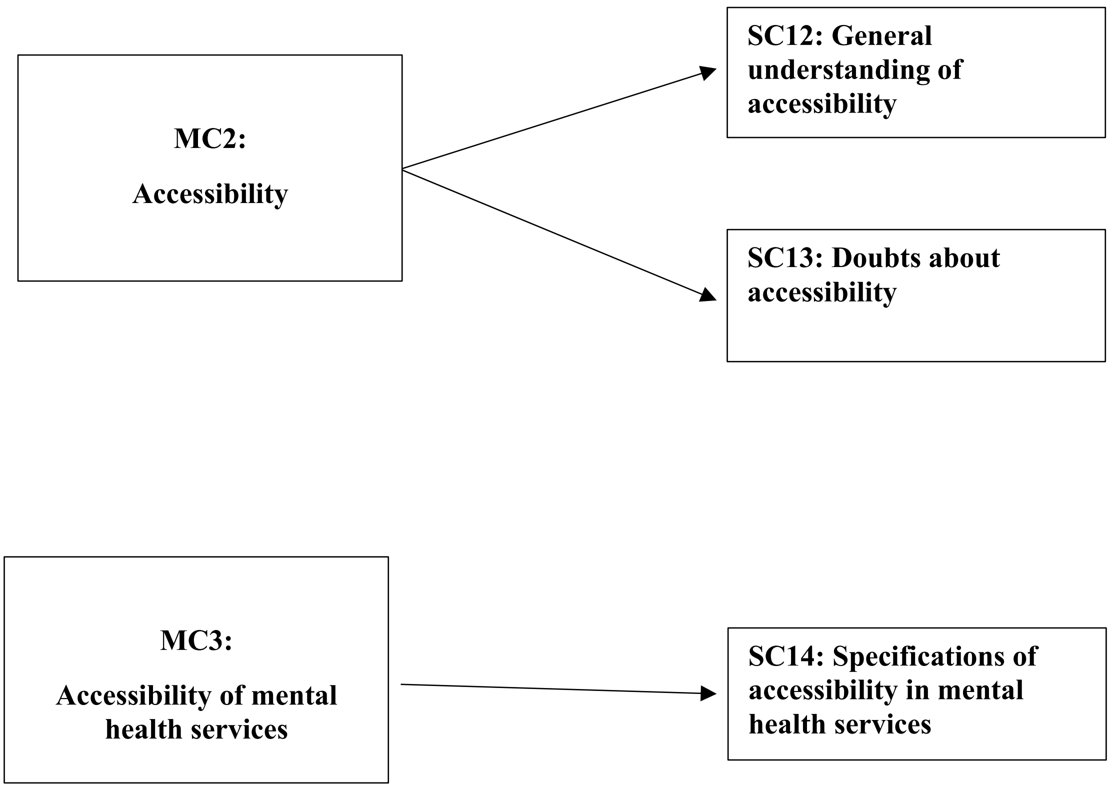

Note: Research question 2: How do professionals and clients describe the accessibility of mental health services for persons with HI or VI, and what are the barriers?

Figure 3

Main category MC4 including subcategories SC15 to SC27

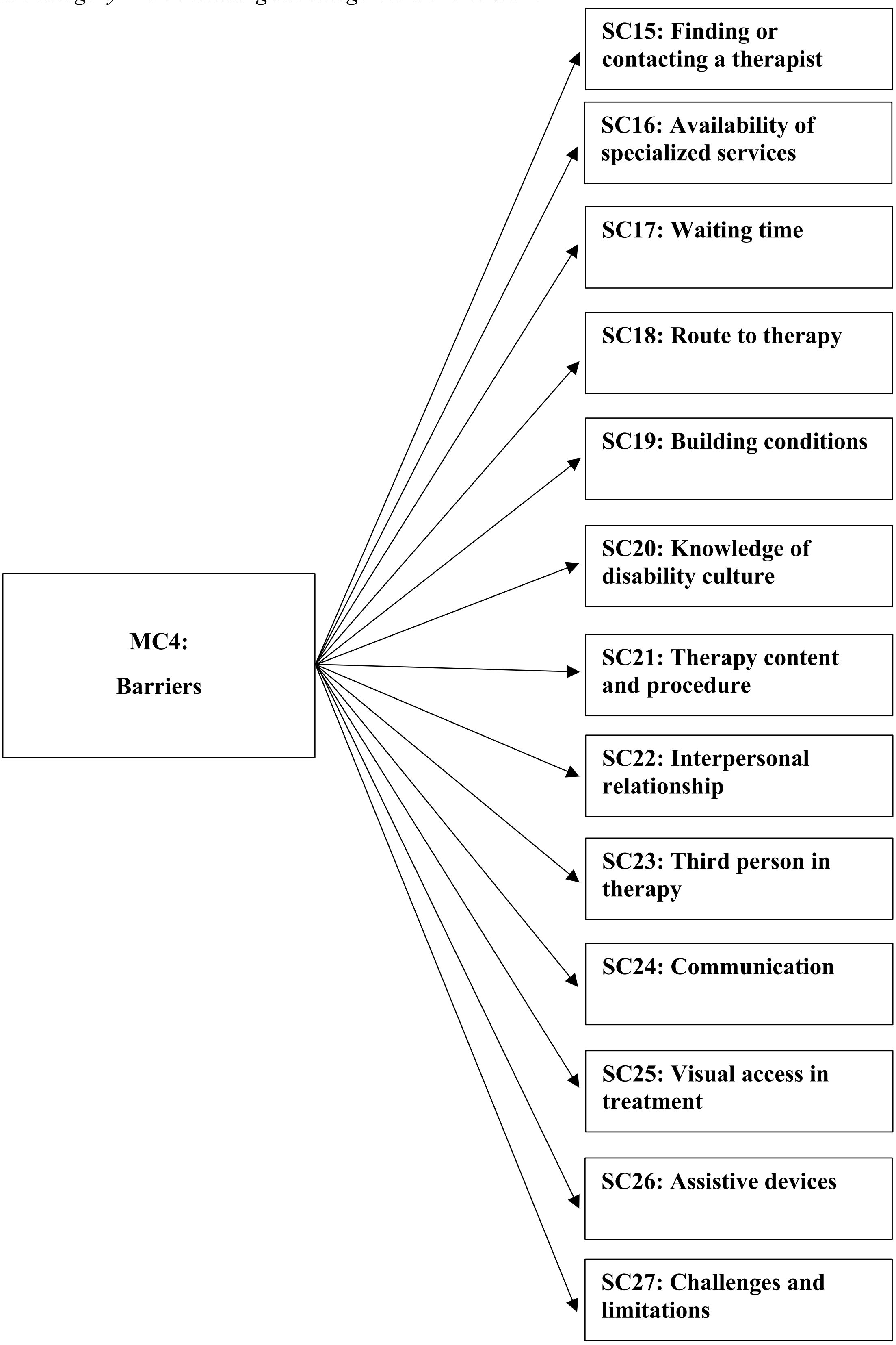

Note: Research question 2: How do professionals and clients describe the accessibility of mental health services for persons with HI or VI, and what are the barriers?

Figure 4

Main category MC5 including subcategories SC28 to SC37

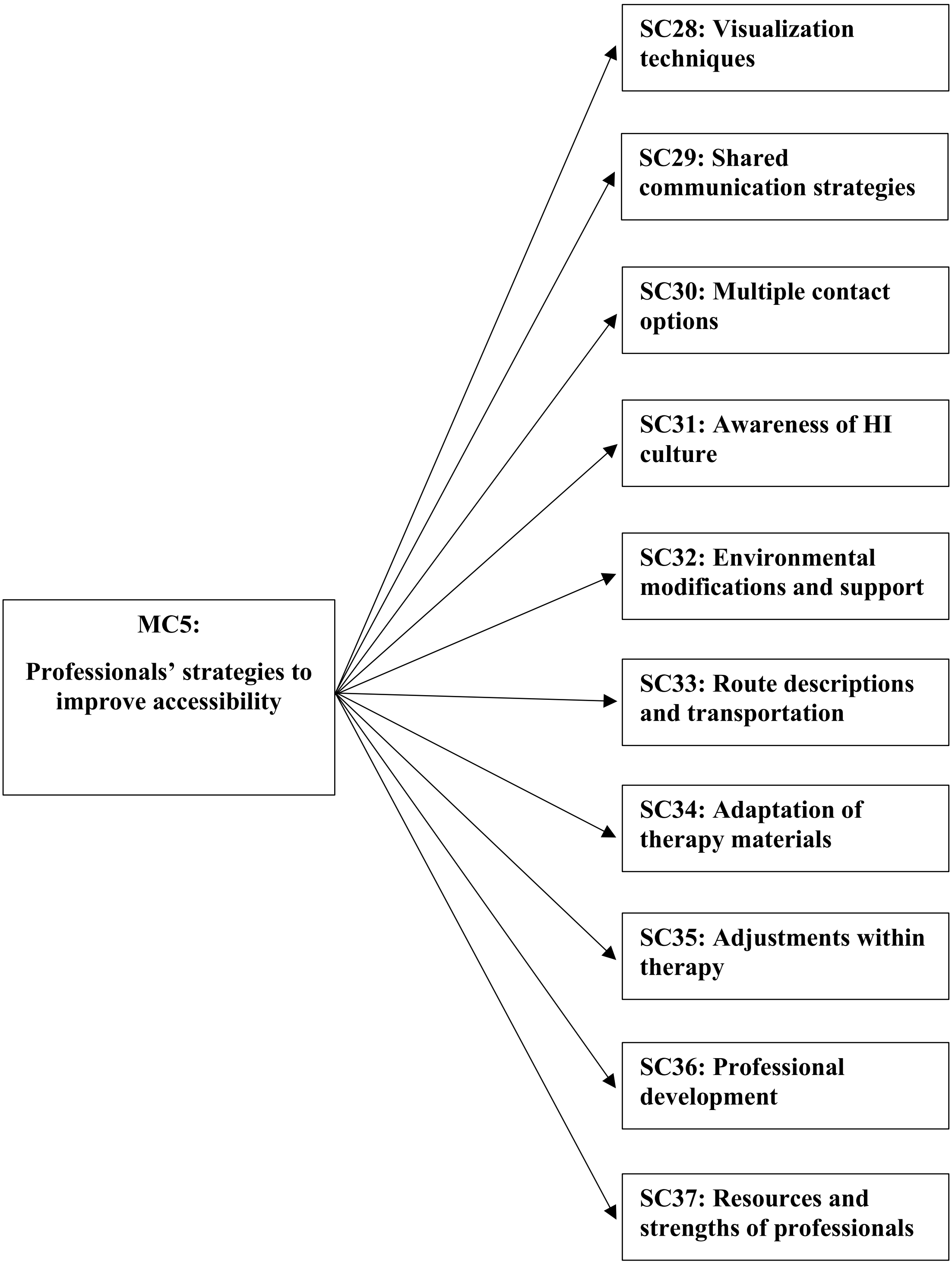

Note: Research question 3: What strategies do professionals and clients apply to improve accessibility in mental health services for persons with HI or VI?

Figure 5

Main category MC6 including subcategories SC38 to SC42

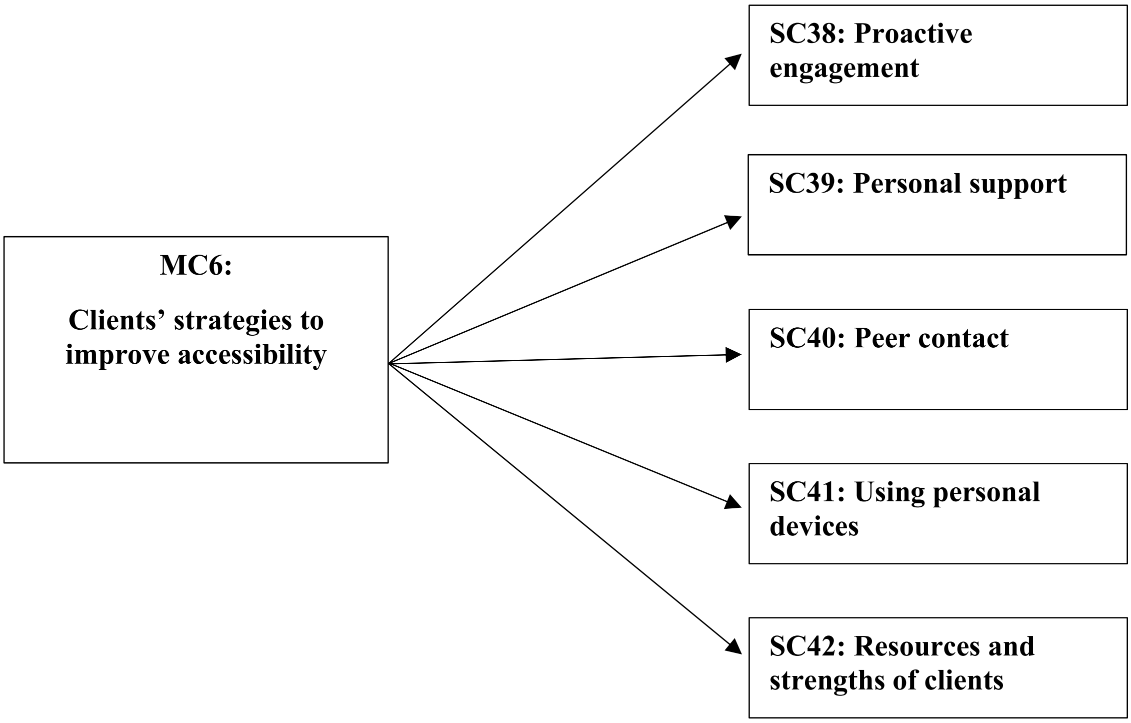

Note: Research question 3: What strategies do professionals and clients apply to improve accessibility in mental health services for persons with HI or VI?

Figure 6

Main category MC7 including subcategories SC43 and SC44

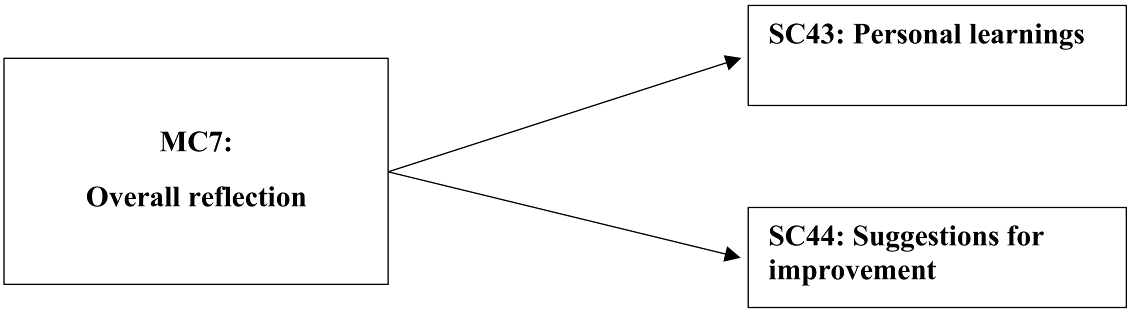

Note: Research question 4: How do professionals and clients reflect on their experiences in mental health services for persons with HI or VI, and what are suggestions for the future?
