# Supplementary material for: Barriers and accessibility‐improving strategies in mental health services for persons with hearing or vision impairments: Perspectives from professionals and clients—A qualitative interview study
Source: Psychol Psychother. 2025 Aug 13;99(1):40–59. doi: 10.1111/papt.70006 (PMC12905524; doi:10.1111/papt.70006)
Supplement: Supplementary file 2 — Table S1 [file PAPT-99-40-s004.docx]

**Supplemental Table 1**

*Demographic data and reported treatment approaches and services of all four groups (N = 58)*

|  | Professionals (HI)  *n* = 19 | Professionals (VI)  *n* = 12 | Clients (HI)  *n* = 14 | Clients (VI)  *n* = 13 |
| --- | --- | --- | --- | --- |
| Gender distribution (female: *n*; %) | 14 (73.68%) | 8 (66.67%) | 12 (85.71%) | 9 (69.23%) |
| Mean age (SD) | 47.66 (9.75)  Range: 31-63 | 51.50 (13.52)  Range: 32-69 | 44.93 (15.04)  Range: 21-71 | 44.77 (13.40)  Range: 26-69 |
|  |  |  |  |  |
| Personal HI or VI (*n;* %) | 4 (21.1%) | 6 (50%) | 14 (100%) | 13 (100%) |
| Treatment approach (professionals) and service utilization (clients):   - Behavioral Therapy - Psychodynamic Psychotherapy - Psychoanalysis - Psychological Counselling - Systemic Psychotherapy - Conversation Psychotherapy - Gestalt Therapy - Traumatherapy - Psychooncology - Eye Movement Desensitization and Reprocessing | 7 (36.8%)  6 (31.6%)  NM  NM  4 (21.1%)  NM  2 (10.5%)  NM  NM  2 (10.5%) | 4 (33.3%)  4 (33.3%)  NM  3 (25%)  2 (16.7%)  NM  NM  NM  NM  NM | 11 (78.6%)  2 (14.3%)  1 (7.1%)  NM  3 (21.4%)  2 (14.3%)  NM  NM  1 (7.1%)  NM | 9 (69.2%)  5 (26.3%)  1 (5.3%)  NM  NM  1 (5.3%)  NM  1 (5.3%)  NM  NM |

*Note: HI = hearing impairments; VI = vision impairments, SD = standard deviation; NM = Not Mentioned.*
