# Supplementary material for: Barriers and accessibility‐improving strategies in mental health services for persons with hearing or vision impairments: Perspectives from professionals and clients—A qualitative interview study
Source: Psychol Psychother. 2025 Aug 13;99(1):40–59. doi: 10.1111/papt.70006 (PMC12905524; doi:10.1111/papt.70006)
Supplement: Supplementary file 3 — Table S2 [file PAPT-99-40-s003.docx]

**Supplemental Table 2**The category system of seven main categories (MCs) for all four groups

| **Code** | **Definition** | **Coding Rule** | **Anchor Example** |
| --- | --- | --- | --- |
| MC1: Providing/accessing mental health services for persons with HI or VI | How professionals started treating persons with HI or VI and how clients found their professionals/ treatment. | Statements and examples describing how professionals initially started to work with clients with HI or VI, as well as how clients established first contact with therapists. | *"Then I searched for therapists on the internet and through the Association of Statutory Health Insurance Physicians.”* (Client_VI) |
| MC2: Accessibility | What professionals and clients generally understand by the term "accessibility". | Statements and examples that subjectively describe the term "accessibility" regardless of the mental health setting. | *"Accessibility means that wherever people need support, the barriers that prevent me from participating should at least be reduced."* (Client HI) |
| MC3: Accessibility of mental health services | What professionals and clients understand by "accessible mental health services". | Statements and examples about the understanding and conception of accessibility in the context of mental health services. Examples from personal therapy experiences only if they are considered generalizable by the individual. | *"So, by 'accessible' I mean that hearing-impaired people have access to the same services as hearing people. For example, that they receive counseling or therapy in sign language, or even basic care, which is, of course, lacking."* (Professional_HI) |
| MC4: Barriers | Barriers that professionals and clients have personally experienced or have generally expected for persons with HI or VI in mental health services. | Descriptions of all areas where professionals and clients have experienced or generally imagine disability-related restrictions within treatment. | *"The psychotherapists I visited at that time were all at least on the upper floor, so they were accessible by some steps. Mine, for example, was in an old building, which was terribly dark."* (Client_VI) |
| MC5: Professionals’ strategies to improve accessibility | What professionals have concretely done to overcome barriers and improve accessibility. | All statements and examples referring to strategies that professionals have specifically applied to counteract disability-related restrictions in mental health services for clients with HI or VI. | *"Well, at the beginning of the conversation, I communicate with the patient who has a hearing impairment: 'Do you understand me? Is this okay? Please let me know if something is not right. Don’t hesitate. Or speak up.' So, I basically clarify this in advance. It's a kind of meta-communication."* (Professional_HI) |
| MC6: Clients’ strategies to improve accessibility | What clients have concretely done to overcome barriers and improve accessibility. | All statements and examples referring to strategies that clients have specifically applied to counteract disability-related restrictions in mental health services. | *"Today, many people already have iPhones or similar devices, where you can send them a worksheet digitally, and then they can read it with VoiceOver on the iPhone, which works quite well."* (Professional_VI) |
| MC7: Overall reflection | How professionals and clients reflect on their previous experiences in mental health services. | All statements and examples that provide a summary of the mental health service experience, including positive and negative aspects and suggestions for improvement, from the perspective of professionals treating persons with HI or VI and clients with HI or VI. | *"Before I became deaf, I was not aware that psychotherapy is not accessible to everyone [...] now I know the full extent, and it is very distressing because it is associated with a lot of suffering."* (Client_HI) |

*Note: HI = hearing impairment; VI = vision impairment.*
