# Supplementary material for: Barriers and accessibility‐improving strategies in mental health services for persons with hearing or vision impairments: Perspectives from professionals and clients—A qualitative interview study
Source: Psychol Psychother. 2025 Aug 13;99(1):40–59. doi: 10.1111/papt.70006 (PMC12905524; doi:10.1111/papt.70006)
Supplement: Supplementary file 4 — Table S3 [file PAPT-99-40-s001.docx]

**Supplemental Table 3**

*Examples for all inductive subcategories (SCs) identified regarding the four perspectives on mental health services for persons with HI or VI*

| Category | Professionals (HI) | Professionals (VI) | Clients (HI) | Clients (VI) |
| --- | --- | --- | --- | --- |
| MC1: Providing/accessing mental health services for persons with HI or VI | | | | |
| SC1: Personal disability | *n* = 4  Quote: "Primarily because of my own experience and to be able to apply my own experiences usefully." | *n* = 6  Quote: "Well, I'm blind myself, so it made sense for me to work with visually impaired and blind people." | N/A | N/A |
| SC2: Professional life | *n* = 9  Quote: "I did my thesis at a school for the deaf and learned about the great need for support." | *n* = 11  Quote: "I believe my first contact was as a student during an internship at a psychosomatic clinic." | N/A | N/A |
| SC3: Literature research | *n* = 3  Quote: "I built my background knowledge with the help of colleagues, specialized literature.” | *n* = 7  Quote: "I also looked it up or checked online." | N/A | N/A |
| SC4: Exchange with persons with HI or VI | *n* = 5  Quote: "I sought contact with persons with hearing impairments, read literature, and also wrote my thesis in this field." | *n* = 8  Quote: "I asked the patient extensively about her experiences, including the diagnosis and the medical aspects." | N/A | N/A |
| SC5: Exchange with specialized professionals | *n* = 6  Quote: "I learned a lot from my instructors during my studies, who were fluent in sign language at a native level." | *n* = 5  Quote: "Through senior physicians and blind specialists." | N/A | N/A |
| SC6: Fascination of sign language | *n* = 10  Quote: “I have always been drawn to sign language. I had nothing to do with  deaf people before.” | N/A | N/A | N/A |
| SC7: Online search | N/A | N/A | *n* = 6  Quote: "I researched it as well. You know, it's easy to find names through the internet." | *n* = 4  Quote: "I then found the phone number online." |
|  |  |  |  |  |
| SC8: Recommendations from professionals | N/A | N/A | *n* = 8  Quote: "Well, initially my primary care physician, with whom I had spoken, referred me to a therapist who quickly assessed my situation to determine for the doctor: 'Does she need therapy or not?'" | *n* = 5  Quote: "I knew I needed help, so my primary care physician gave me a referral. Then my search continued through the health insurance company." |
| SC9: Word of mouth | N/A | N/A | *n* = 3  Quote: "I find that email works quite well, but I mostly find therapists through word of mouth. Yes, it's about knowing each other. And that way, you connect more quickly." | *n* = 2  Quote: "A friend referred me to the city's psychosocial service. The social worker there recommended that I undergo therapy and called my current therapist for me." |
| SC10: Contact by phone | N/A | N/A | N/A | *n* = 4  Quote: "I obtained a list from the health insurance company and called through it." |
| SC11: Reasons for beginning therapy | N/A | N/A | *n* = 13  Quote: "Uncertainty. I just didn't know exactly what to expect or how the whole process would go." | *n* = 7  Quote: "I expect a therapist to interact with me in an empathetic yet emotionally detached manner, because I want to have a question or challenge resolved or to get an idea of how I can solve it myself." |
| MC2: Accessibility | | | | |
| SC12: General understanding of accessibility | *n* = 16  Quote: "Equal opportunities in everyday life, equal access (e.g., to public offices), reduction of physical barriers." | *n* = 11  Quote: "[...] equal access to the same offers and without any obstacles. So that I can manage and carry it out on my own and don't necessarily have to be taken by the hand." | *n* = 9  Quote: "Accessibility means that wherever people need support, the barriers that prevent me from participating should at least be reduced." | *n* = 4  Quote: "By accessibility, I mean removing the barriers that prevent people from participating in life and everyday life." |
| SC13: Doubts about accessibility | NM | *n* = 2  Quote: "First of all, I believe that true barrier-free accessibility hardly exists. It's more accurate to say it should be barrier-reduced. What do I mean by that? That someone with a disability should not have significantly more hurdles to overcome." | NM | *n* = 1  Quote: "I don't believe that complete accessibility truly exists, and I don't think it's necessary either, as disability is, after all, a condition. Every person with a disability is different, and there will always be some stumbling blocks." |
| MC3: Accessibility of mental health services | | | | |
| SC14: Specifications of accessibility in mental health services | *n* = 17  Quote: "[...] communicative accessibility, individual and group therapy directly in sign language." | *n* = 9  Quote: "I think it has various aspects, such as accessibility via public transportation, accessible work materials, things like that." | *n* = 7  Quote: "[...] being able to choose the preferred mode of communication yourself. So that [...] one can communicate in the same way as a hearing person would." | *n* = 4  Quote: "You don't have to print it out first and get help or assistance, you can edit the form yourself." |
| MC4: Barriers (expected/experienced) | | | | |
| SC15: Finding or contacting a therapist | *n* = 6 / *n* = 8  Quote: "Some therapists are only available by phone." | *n* = 5 / N/A  Quote: "The access itself: 'How does one even get into therapy?' Older people, they can't read, and they don't have any screen reader or anything like that. So, it's not just about finding out about available services, but also actually getting there." | *n* = 4 / *n* = 10  Quote: "If I had one wish, it would be for contact via email." | *n* = 6 / *n* = 8  Quote: "After they told me in the hospital that I should go to psychosomatics, I had three consultations on the same day. At the clinic in [location 1], I would have had to wait several weeks, so it would have taken longer. And the clinic in [location 2] said, 'We can't take you because of your blindness.' |
| SC16: Availability of specialized services | *n* = 16 / *n* = 4  Quote: "There are only a few options available for patients who are searching." | *n* = 4 / *n* = 5  Quote: "It depends, but what is very, very rare, and in some regions does not exist at all, is someone with experience in this field." | *n* = 5 / N/A  Quote: "There are hardly any therapeutic services available... Neither outpatient nor inpatient, the latter are almost non-existent.”FormularbeginnFormularende | *n* = 3 / *n* = 4  Quote: "Disability is not addressed at all in therapy training. Dealing with intellectual disabilities, hearing impairments, or other issues just doesn't come up. It seems like therapy is only for non-disabled people." |
| SC17: Waiting time | *n* = 2 / N/A  Quote: "There is a long wait time for patients—an average of 3 months from the initial consultation to the first therapy session." | *n* = 2 / N/A  Quote: "In general, I would say that the usual waiting times for psychotherapy are simply inadequate because people with visual impairments often find themselves in more urgent situations." | *n* = 3 / *n* = 6  Quote: "Exactly. So, my therapist was initially a bit concerned that the 2 months might be too long; she felt really sorry about it. But now, when I hear how long others wait for a therapy place, it seems like a storybook situation." | N/A / *n* = 4  Quote: "And then a psychotherapist opened a practice in our town, so I called, and I was put on his waiting list. I called several times back then. I think I had to wait for about two years." |
| SC18: Route to therapy | *n* = 1 / N/A  Quote: "There are patients who have to travel a long distance for therapy, especially in rural areas." | *n* = 8 / *n* = 2  Quote: "Regarding accessibility from the subway to the practice: Getting there in the first place is a hurdle and affects participation." | *n* = 2 / N/A  Quote: "There are access limitations, such as waiting times, long distances, application procedures, and knowing whom to contact." | *n* = 2 / *n* = 1  Quote: "For those of us with visual impairments, public transportation is really difficult." |
| SC19: Building conditions | NM | *n* = 7 / *n* = 1  Quote: "It’s possible that a building is designed in such a way that a patient has difficulty accessing certain areas. Finding their way around the building and knowing the layout. That's where I think there are many barriers." | *n* = 1 / *n* = 9  Quote: "That rooms are echoey, meaning they are not acoustically treated." | *n* = 2 / *n* = 6  Quote: "Some clinics are problematic in terms of their structural conditions. There's too little lighting, incorrect lighting, no Braille, and no high-contrast signage." |
| SC20: Knowledge of disability culture | *n* = 11 / N/A  Quote: "Poor therapeutic care due to a lack of knowledge about hearing impairments and Deaf culture, as well as limited sign language proficiency. There are only a few therapists with expertise in this field." | *n* = 8 / *n* = 6  Quote: "I could imagine that for a therapist who has no prior experience with visual impairments, it might be challenging at first, especially with more severe limitations. They would need to empathize with the person’s life situation a bit more." | N/M | *n* = 4 / *n* = 7  Quote: “They had an image in their minds of how a blind person lives that didn’t match *who I actually am at all.”* |
| SC21: Therapy content and procedures | N/A / *n* = 8  Quote: "Diagnosis is challenging for patients who do not have a good command of German or who only know the written language. As a result, working with homework in CBT is often not feasible with deaf patients." | *n* = 5 / *n* = 7  Quote: "If people here had to go to the trauma unit, I think they’d be in a tough spot. They use a lot of worksheets in behavioral therapy that have to be filled out. Several things need to be completed and checked daily. Diagrams are everywhere. There’s nothing there for blind and visually impaired people. They’d be pretty much left behind." | N/A / *n* = 12  Quote: "However, with this EMDR, you sit there with your eyes closed in front of the light, and she [therapist] talks. And that was sometimes difficult for me as well. Because at that time I didn’t have the cochlear implant yet. And then, I was very dependent on lip-reading, and I noticed that this was a problem." | *n* = 3 / *n* = 8  Quote: "Worksheets that we can't read, that we can't fill out, and some anamnesis forms that we can't complete." |
| SC22: Interpersonal relationship | *n* = 7 / *n* = 11  Quote: "Many fellow therapists might have reservations—physical aspects and their own fears are projected onto the patient." | *n* = 11 / n = 4  Quote: "The biggest difficulties, in my view, are on the emotional level, such as prejudices, insecurities, and also defensive reactions from colleagues who have no experience with this." | *n* = 3 / *n* = 9  Quote: "The question is, how much do people with hearing impairments trust a hearing psychotherapist? [...] And that alone makes it difficult." | *n* = 9 / *n* = 6  Quote: "Many people can't understand: 'You seem to see normally, you walk normally—so what's wrong with you?'" |
|  |  |  |  |  |
| SC23: Third person in therapy | N/A / *n* = 14  Quote: "For deaf persons, therapy without a sign language interpreter is more comfortable. The trust in interpreters is often lacking because they can't be sure if what they said is being translated accurately. They feel excluded again. Additionally, there is a lack of eye contact with the therapist." | NM | N/A */ n* = 1  Quote: "If I really wanted to understand everything the therapist says, I would need a speech-to-text interpreter. But I wouldn’t want a third person in the therapy. So, I have to choose to understand less in order to maintain my privacy." | N/A / *n* = 6  Quote: "It’s uncomfortable having to fill out the questionnaires with the help of someone who can see. For them, it was obvious that my father, as my companion, would fill out the questionnaire. But I don’t want to fill out a psychological questionnaire with my father." |
| SC24: Communication | *n* = 11 / *n* = 9  Quote: "Poor lip-reading, difficult for people with hearing impairments to understand." | NM | *n* = 9 / *n* = 14  Quote: "It was very, very exhausting to listen. Hearing is mental work, and it’s just tiring to listen to someone who speaks very softly, someone you constantly have to remind that you have difficulties in hearing and can’t always follow along." | N/A / *n* = 2  Quote: "I sometimes cried, and then I looked at a blacked-out face. That was really frustrating for me because I couldn't see her [therapist] facial expressions." |
|  |  |  |  |  |
|  |  |  |  |  |
| SC25: Visual access in treatment | NM | *n* = 7 / *n* = 3  Quote: "In diagnostics, such as with questionnaires, one could say: 'Therapists can go through it with the patient.' However, this means patients lose valuable session time. Other patients who can fill out the questionnaires at home have the full 50 minutes to address other topics." | NM | *n* = 2 / *n* = 4  Quote: "I try to focus on something to recognize it, and then I end up looking strained or angry. I have to point that out." / "There were situations in communication where I misinterpreted the therapist's facial expressions." |
|  |  |  |  |  |
|  |  |  |  |  |
| SC26: Assistive devices | NM | *n* = 2 / *n* = 2  Quote: "It's always very problematic with screen readers. They usually don't explain graphics." | N/A / *n* = 7  Quote: "Yes, there were occasional situations where the battery was running low or there were technical problems, which sometimes disrupted the conversation. When this happened during a particularly sensitive or intense therapy session, it was very disruptive." | N/A / *n* = 2  Quote: "Assistive devices are unfortunately not used at all by such institutions [clinics]." |
| SC27: Challenges and limitations | *n* = 13  Quote: "We have observed that people with language deprivation are very difficult to reach because they generally have limited knowledge, limited education, and few linguistic resources." | *n* = 9  Quote: "When I think about my first patients, I was rather unsure: ‘Am I up to this? Can I do this?’ Because the Medical Association, the Association of Statutory Health Insurance Physicians, and before that, when I worked in psychiatry at the hospital, they were all convinced that I couldn’t do it. And so, I always had this question in the back of my mind: ‘Can I really do this?’ I think I can do it, I do it a little differently, but I’m proving them wrong. This created a kind of pressure: ‘I have to prove that I can do it.’" | *n* = 2  Quote: "Well, it was definitely a limitation for me not to be able to fully participate in all the exercises or to experience them in the way I would have liked. I definitely reached my limits there." | *n* = 4  Quote: "You already always feel like you can't be part of things anymore, like you don't really belong to this world. And then therapy facilities basically reinforce that by rejecting you. That was the worst part for me—this prevented sense of belonging." |
| MC5: Professionals’ strategies to improve accessibility | | | | |
| SC28: Visualization techniques | *n* = 8  Quote: "So what has worked well so far is the visualization of things. For example, when I ask, 'What do you think he’s thinking?'—they didn’t understand that. But then representing it with coffee cups, objects, or Playmobil figures, like, 'This is the father, and this is the mother. What is he thinking? What is she thinking?' That’s one point: visualization. Then, also simplification—I try to think about how I can phrase things even more simply, even more clearly. But I have to say that experience is a really important factor in making this adaptation process successful. Visualization, simplification." | NM | NM | N/M |
| SC29: Shared communication strategies | *n* = 10  Quote: "Sometimes we even make something up ourselves. If we know what we want to say and they don’t know a sign for it, then we invent one. Because if I know that this word will come up again, we need a sign for it." | NM | *n* = 9  Quote: "They made an effort to implement what I said I needed for communication. For example, they naturally waited for me to finish with my technology and hand them the microphone before starting to speak. In one-on-one conversations, they made an effort to speak clearly, slowly, and directly with me." | NM |
| SC30: Multiple contact options | *n* = 8  Quote: "Yes, the ability to make contact has really improved because nowadays there’s almost always the option to use email and not just phone calls. Over the years, this has truly expanded, and now there’s also video calls..." | NM | *n* = 9  Quote: "Yes, and then there's the thing with the phone, that you write emails or WhatsApps instead." | NM |
| SC31: Awareness of HI culture | *n* = 10  Quote: "When did a person become deaf? Did they identify as hearing or deaf? A lot of psychological distress depends on this, and it can also be reduced if the issue of identity is addressed." | NM | NM | NM |
| SC32: Environmental modifications and support | *n* = 9  Quote: "That could be true, yes, for example, if the treatment room echoes too much. There are rooms where the sound bounces back from the walls because of the furniture or because there's no carpet or anything to absorb the sound, so it becomes echoey—it clatters. And that's particularly difficult for people with a cochlear implant sometimes." | *n* = 9  Quote: "There are markings on the floor throughout the rooms here. So, whether using a white cane or just being attentive, you can notice them as you walk over them. You can walk down the corridors without bumping into the walls on either side. I believe there's also a model here where you can feel where you are in the building and which room number is nearby. So, such support is available." | *n* = 11  Quote: "Suggestions or ideas were offered on how to reduce the barrier, if not eliminate it entirely. For instance, good lighting, minimizing background noise, writing things down, etc." | *n* = 7  Quote: "She [therapist] was very open to change and to suggestions. At one point, she moved to a different practice and actually made sure that there were direct paths, that it was well-lit, and she even placed her chairs differently so that, for example, the window was to the side." |
| SC33: Route descriptions and transportation | NM | *n* = 8  Quote: "I am well-prepared to work with this group of people. I can describe to anyone how to get to my practice using public transportation. I can describe the walking route in such a way that even a blind person can manage it." | NM | *n* = 4  Quote: "I was fortunate to receive a taxi ride thanks to the designation of blindness on my disability ID." |
| SC34: Adaptation of therapy materials | *n* = 3  Quote: "I started adapting my own materials, taking things I like to use, that I enjoy working with, but that are too text-heavy or too difficultly or awkwardly phrased, and then I simply reworked them with either images or simpler language." | *n* = 9  Quote: "When I needed something for the patients, I provided it to them either in Braille, large print, or on a CD." | NM | *n* = 3  Quote: "Even though the questionnaires were long, we filled them out together. I really appreciated that, especially when they reassured me that I didn’t need to feel awkward about us completing them together." |
| SC35: Adjustments within therapy | *n* = 9  Quote: " With the instructions, the deaf participants can see which exercises we’re doing, and during the relaxation part where you’re supposed to close your eyes, they close their eyes. Then, at the end of the relaxation time, the therapist gently touches the first person on the knee, who then touches the next person on the knee, and so on, until everyone is back, and then we proceed with the next exercise. This is an adaptation we’ve made in this context, for example." | *n* = 8  Quote: "You just have to see how the client is wired. You discuss it: 'What works best for you? Would you like something tactile, or is it enough if I explain it to you?' 'Should I record it on a CD?' You sometimes need to talk about it and adjust the methods accordingly." | *n* = 9  Quote: "She [therapist] notices that I ask for clarification much more often than before, and she can tell that I’m much more relaxed when we have video sessions. When I stay at home, she doesn’t have to wear a mask in her office. That’s one thing. The other is that without the hearing system, it wouldn’t work at all." | *n* = 4  Quote: "My therapist in group therapy did an excellent job. When something involving facial expressions happened that was relevant to the context, she described it." |
| SC36: Professional development | *n* = 12  Quote: "A lot of intervision, a lot of supervision, a lot of exchange with others, ideally with hearing-impaired counselors, social workers, and social educators. A lot of literature. There isn’t much literature, so it’s like mandatory sessions." | *n* = 9  Quote: "Exactly, intervisions are groups. We meet and discuss current topics. We exchange ideas and provide input: 'Have you tried this?' and 'I would start with this.' It's just an exchange of ideas." | N/A | N/A |
| SC37: Resources and strengths of professionals | *n* = 17  Quote: "I always have to smile when I see someone with a migration background in the community of practitioners, because it’s often hinted at that the motivation behind it is something like, ‘Yeah, I know what it’s like.’ And then you smile at each other, knowing why you ended up in this field. If someone doesn’t have any direct professional or personal connections with deaf or hearing-impaired people, then it’s more like, ‘I was somewhere abroad and didn’t know how to communicate.’" | *n* = 10  Quote: "I believe [...] that it was easier for people to put their disability and the whole topic on the table because I am personally affected." | *n* = 10  Quote: "That they stay calm when maybe 3, 4, or 5 questions are asked because something just wasn’t understood. And also, not start rephrasing it five different ways, but just try to repeat it as it was. Of course, as far as one can still remember exactly. Sometimes it's hard to say something exactly the same way again, but if you start changing 5-6 words, then it’s sometimes just slightly different. That’s definitely a strength." | *n* = 10  Quote: "On the one hand, she [therapist] was able to convince me that it’s not absolutely necessary for the person I’m talking to that he or she knows every detail of my condition — they don’t need to be my second eye doctor to understand the issues that come with it. Yes, she convinced me of that quite well. And also, to become more consciously aware of life in a different way." |
| MC6: Clients’ strategies to improve accessibility | | | | |
| SC38: Proactive engagement | *n* = 12  Quote: "When I introduce myself, I ask (supported by signs): How should I speak so that you can understand me well?" | *n* = 11  Quote: "This emerged in conversations with the patients: 'Where do you need help?' 'What is life like with a disability?' The main focus was on the acceptance of disability." | *n* = 8  Quote: "Ideas or suggestions were brought up on how to not eliminate the barrier, but at least reduce it somewhat. For example, good lighting, no background noise, writing things down, etc." | *n* = 9  Quote: "I told them, 'Listen, I am visually impaired. Please provide me with a room that is bright and well-lit.'" |
| SC39: Personal support | *n* = 5  Quote: "But it’s a kind of reassurance, sometimes, when a family member comes along, who either acts a bit like a translator or is actually more focused on spoken language. Because at the beginning, they might not yet know how well I can sign, whether it will work, whether communication without an interpreter will actually work, which is completely understandable." | *n* = 7  Quote: "The nursing staff here at the clinic is also well-organized. [...] The nursing staff takes it [therapy materials] and reads it aloud to the patients." | *n* = 5  Quote: "Exactly, it works with interpreters, even for language barriers like foreign languages. It works because people take it into consideration. So why shouldn’t it work with hearing impairments? Yes, exactly." | *n* = 6  Quote: "I have my partner, who drives me to therapy and picks me up afterward." / "A friend of mine called the therapist on duty and explained to her that blindness is really nothing to be afraid of." |
|  |  |  |  |  |
| SC40: Peer contact | NM | *n* = 5  Quote: "If there is a good self-help group available somewhere, it is definitely useful." | NM | NM |
| SC41: Using personal devices | *n* = 10  Quote: "FM system. I don’t have one in my practice, but in the clinic, if necessary, yes. These systems are very, very expensive. And in one-on-one conversations, they are usually less necessary. And those who need it usually have their own amplification device." | *n* = 6  Quote: "Today, many people have an iPhone or something similar. You can send a worksheet digitally, and they can read it with VoiceOver on their iPhone. It works quite well." | *n* = 11  Quote: "If I hadn’t had the technology that I personally use, I wouldn’t have been able to do the therapy." | *n* = 11  Quote: "I always carry a strong flashlight with me to make things visible." / "Computers weren’t allowed in the clinic, but I had one." |
| SC42: Resources and strengths of clients | *n* = 14  Quote: "Authenticity and directness. If you understand that and don't take it personally, then it’s okay—it’s a resource, I think. You just have to learn to deal with this directness, like when they say, 'You used to dye your hair. It was much nicer. Your gray hair just makes you look old.'" | *n* = 10  Quote: "[…] when there is a certain level of motivation, when they come here and genuinely want to achieve something, then we can support them very well." | *n* = 9  Quote: "I really appreciated the option to just say, 'Okay, I’m turning off my hearing aids now,' when it got too loud. I found that very helpful, especially when I was feeling really stressed. Particularly in occupational therapy, when everyone was working intensely, for example." | *n* = 8  Quote: "I've always managed to help myself through hard work and effort, using a screen reader, magnifying glass, or, if necessary, with support from my family to make sure I submitted everything dutifully and on time." |
| MC7: Overall reflection | | | | |
| SC43: Personal learnings | *n* = 8  Quote: "The wish is for society to be more informed about Deaf culture and sign language, as there are many misconceptions. Specifically, therapists can learn that there is a strong cultural identity and a tight-knit community, which can make a significant positive contribution to one’s life." | *n* = 11  Quote: "It’s not easy work, for either side, because you inevitably confront existential questions. But when you manage to address them together, it’s definitely enriching for the patient and, to some extent, also for the therapist." | *n* = 14  Quote: "Before I became deaf, I wasn’t aware that psychotherapy isn’t accessible to everyone […] now I know the full extent, and it’s very distressing because it’s tied to so much suffering. The system is simply […] not designed […] to remove additional barriers and […] to address individual needs." | *n* = 6  Quote: "In outpatient therapy, I see a lot of positive aspects (...). In the inpatient sector, there's an enormous amount of work to be done. The barrier is essentially one hundred percent because many clinics don't admit people with visual impairments at all." |
| SC44: Suggestions for improvement | *n* = 17  Quote: "...offer a module on sign language as part of therapy training." | *n* = 11  Quote: "And I do believe that the quality of therapy […] would be more promising […] if, for example, topics related to disabled patients played a greater role in therapist training." | *n* = 12  Quote: "So, yes. Ideally, I would like to see more therapists spread across Germany. Or for therapists to offer more services like — 'We also have a speech-to-text interpreter available' — or to openly show that they've thought about communication and that they will find a way to make it work. I've noticed that with most therapists, I always check their website." | *n* = 10  Quote: "(...) that even more people receive additional training to address the specific needs of individuals who are blind or visually impaired." |

*Note: HI = hearing impairment; VI = vision impairment; N/A = Not Available (e.g., regarding topics within the interview guide); NM = Not Mentioned.*
