# Supplementary material for: Barriers and accessibility‐improving strategies in mental health services for persons with hearing or vision impairments: Perspectives from professionals and clients—A qualitative interview study
Source: Psychol Psychother. 2025 Aug 13;99(1):40–59. doi: 10.1111/papt.70006 (PMC12905524; doi:10.1111/papt.70006)
Supplement: Supplementary file 5 — Appendix S1 [file PAPT-99-40-s005.pdf]

Supplemental Material 5

The interview guides were originally developed and conducted in German. For the purpose of this supplemental file, they have been translated into English by the research team. The translation was designed to preserve semantic and contextual equivalence; however, it has not undergone formal validation. Where necessary, wording has been adapted to ensure clarity and accessibility for an international audience.

Sections

Interview Guide for Mental Health Professionals (German Original) 2
Interview Guide for Mental Health Professionals (English Translation) 11
Interview Guide for Clients (German Original) 20
Interview Guide for Clients (English Translation) 29

Note on terminology:

In the interview guide for mental health professionals, we use the term "psychotherapist" as a general reference. When the interview participants had a different professional background (e.g., as psychological counselors, psychologists, or others), we adapted the professional title used during the interview to reflect their specific role.

Interview Guide for Mental Health Professionals (German Original)

Interviewkapitel

1. Einleitung & Instruktion
2. Kurzfragebogen
3. Hauptteil
    3.1 Block A: Anbieten von Psychotherapie/Beratung für Menschen mit einer Hör-/ Sehbeeinträchtigung
    3.2 Block B: Barrierefreiheit & Barrieren
    3.3 Block C: Bewältigungsstrategien & Therapiegestaltung
    3.4 Block D: Reflexion
4. Ausklang & Verabschiedung
5. Post Scriptum Eindruck

1. Einleitung & Instruktion

Erst einmal vielen Dank, dass Sie die Zeit und das Interesse aufgebracht haben, mir heute einige Fragen zu beantworten. Bevor wir in das Interview einsteigen, möchte ich Ihnen kurz etwas über den Hintergrund des Interviews erzählen und Ihnen einige Informationen zum Interviewverlauf geben, damit Sie sich ein genaueres Bild davon machen können, was Sie erwartet.

Sinn und Zweck des Interviews

Also: Worum soll es nun in dem folgenden Gespräch gehen? Wie schon vorab erwähnt, möchte ich Ihnen einige vertiefende Fragen dazu stellen, wie barrierefrei Sie als erfahrene(r) Psychotherapeut*in die Psychotherapie von hör-/sehbeeinträchtigten Menschen erleben. Hierbei interessieren mich besonders Ihre eigens gemachten Erlebnisse und Erfahrungen in diesem Bereich.

Was bedeutet das nun konkret für unser Interview – also für meine Fragen und Ihre Antworten?

Es bedeutet, dass während des gesamten Interviews Ihre persönlichen und ganz subjektiven Meinungen Mittelpunkt des Gesprächs sein werden. Folglich gibt es keine

richtigen oder falschen Antworten. Jeder Mensch vertritt schließlich unterschiedliche Meinungen. Von daher können Sie frei und ungezwungen alles berichten, was Ihnen zu der jeweiligen Frage in den Sinn kommt.

Interviewleitfaden

Die Fragen, die ich Ihnen stellen möchte, habe ich für mich schriftlich festgehalten. Ich werde Zwischendurch in meine Unterlagen reinschauen und Notizen machen. Das dient einfach meiner eigenen Orientierung, soll Sie aber nicht stören oder verunsichern.

Aufnahme

Da es mir unmöglich ist, dieses Gespräch vollständig mitzuschreiben, möchte ich es – mit Ihrer Zustimmung versteht sich – aufnehmen. Das hat den Vorteil, dass ich meine volle Aufmerksamkeit auf unser Gespräch lenken kann. Während des Interviews gibt es für Sie immer die Möglichkeit, mir Antworten, die gelöscht werden sollen, mitzuteilen. Das ist bei dem von mir verwendeten Aufnahmegerät hinterher einfacher, als während des Interviews die Aufnahme zu stoppen, da in diesem Falle unterschiedliche Dateien im Gerät erstellt werden. Wenn Sie möchten, lasse ich Ihnen gerne ein verschriftlichtes Exemplar unserer Unterhaltung zukommen.

Dauer

Für das Interview sind ca. 60 – 90 Minuten eingeplant. Sollten Sie während des Interviews eine Pause benötigen, dann geben Sie mir gerne Bescheid. Während dieser Pause würde das Aufnahmegerät gestoppt werden.

Vertraulichkeit und Anonymität

Nun noch einige Hinweise zur Anonymität Ihrer Aussagen: In anderen Interviews hat es sich gut bewährt, dass die einzelnen Teilnehmer*innen zur Kennzeichnung ihrer Person einen Codenamen wählen. So können Sie Ihre Aussagen später erkennen, wohingegen für Dritte nicht ersichtlich ist, welche Person sich hinter der jeweiligen Aussage verbirgt.

Welchen Codenamen möchten Sie für sich wählen?

Haben Sie bis hierin Fragen zum Interview?

Dann beginne ich jetzt mit der Aufnahme und mit dem Interview:

2. Kurzfragebogen

Zu Beginn des Interviews würde ich Ihnen gerne ein paar allgemeine Fragen stellen.

1. Wie alt sind Sie?
2. Welchem Geschlecht fühlen Sie sich zugehörig?
3. Welche Staatsangehörigkeiten besitzen Sie?
4. Zu welcher Konfession/Glaubensrichtung zählen Sie sich?
5. Was ist Ihr höchster Bildungsabschluss? (auch akademische Titel, falls dieser vorhanden ist)
6. Haben Sie selbst eine Hör-/Sehbeeinträchtigung?
    - Wenn Ja: Wie lautet die Diagnose Ihrer Hör-/Sehbeeinträchtigung?
    - In wieweit beeinträchtigt diese Diagnose Ihr Hörvermögen in dB /ihr Sehvermögen (Visus) in Prozent?
    - Haben Sie einen eingetragenen Grad der Behinderung in einem Schwerbehindertenausweis?
        - Wenn Ja: Wie hoch ist Ihr Grad der Behinderung?
        - Wenn ein Schwerbehindertenausweis vorhanden ist: Wissen Sie welche Merkzeichen in Ihrem Schwerbehindertenausweis eingetragen sind? (Zum Beispiel: „Gl“ für gehörlos / „Bl“ für blind, etc.)
    - Ist Ihre Hör-/Sehbeeinträchtigung angeboren oder im Laufe Ihres Lebens aufgetreten?
        - Wenn im Laufe des Lebens aufgetreten: Wie alt waren Sie, als sich Ihre Hör-/Sehbeeinträchtigung zuerst bemerkbar machte?
    - Wie war der Verlauf Ihrer Hör-/Sehbeeinträchtigung nach dem ersten Auftreten? (Zum Beispiel: stabil, verbessert, verschlechtert, etc.)
    - Haben Sie neben Ihrer Hör-/Sehbeeinträchtigung noch weitere eingetragene Diagnosen? Wenn ja, welche?
7. Welcher aktuellen beruflichen Tätigkeit gehen Sie nach?
    - Wie lange arbeiten Sie bereits als Psychotherapeut*in? (in Monate/Jahre)
    - In welchen psychotherapeutischen Therapieformen sind Sie ausgebildet? (Zum Beispiel: kognitive Verhaltenstherapie)

- In welchem Arbeitsumfeld üben Sie Ihre Tätigkeit als Psychotherapeut*in aus? (Zum Beispiel: eigene Praxis, Einzel- oder Gemeinschaftspraxis, stationäre Therapie in einer Klinik, teilstationäre Therapie in einer Tagesklinik, etc.)
- Behandeln Sie ausschließlich privat oder gesetzlich krankenversicherte Patient*innen?

8. Wie viele Patient*innen mit einer Hör-/Sehbeeinträchtigung haben Sie in der Regel? (Im Verhältnis zur Gesamtanzahl an Patient*innen)
   - Wie viele Menschen mit einer Hör-/Sehbeeinträchtigung haben Sie in Ihrer gesamten Laufbahn als Psychotherapeut*in bereits behandelt?
   - Wie lange arbeiten Sie nun schon mit hör-/sehbeeinträchtigten Patient*innen? (Seit Monaten, Jahren)

3. Hauptteil

3.1 Block A: Anbieten von Psychotherapie/Beratung für Menschen mit einer Hör-/ Sehbeeinträchtigung

Einstiegsfrage: Nun haben Sie mir bereits von einigen Informationen über Ihre Arbeit als Psychotherapeut*in berichtet. Gerne würde ich mehr über Ihre persönlichen Erfahrungen als Psychotherapeut*in in der psychotherapeutischen Arbeit mit hör-/sehbeeinträchtigten Patient*innen sprechen. Versuchen Sie sich doch einmal daran zu erinnern, wie Sie dazu gekommen sind, Menschen mit einer Hör-/Sehbeeinträchtigung zu behandeln. Erzählen Sie doch einmal, wie das bei Ihnen war.

(Hinweis für Interviewer: Pause nach der Einstiegsfrage)

Mögliche Themen/Nachfragen in Block A:

- *(Hinweis für Interviewer: Wenn mehrere Erfahrungen vorhanden sind, sprechen Sie bitte über das, was Ihnen gerade in den Sinn kommt, worüber Sie sprechen möchten)*
- Was waren Ihre ersten Berührungspunkte mit dem Thema „Psychotherapie von Menschen mit einer Hör-/Sehbeeinträchtigung“? Geben Sie gerne Beispiele an.
- Wie sah Ihr Weg hin zu einer Psychotherapie von hör-/sehbeeinträchtigten Menschen aus?
- Woher haben Sie Ihr Wissen über die verschiedenen Hör-/Sehbeeinträchtigungen bezogen? Geben Sie gerne Beispiele an.

- Wie waren Ihre eigenen Erwartungen als Psychotherapeut*in an die Psychotherapie von hör-/sehbeeinträchtigten Menschen vor Ihrer ersten Behandlung? (Zum Beispiel: Wünsche und/oder Vorbehalte)
- Wie hat sich Ihre Herangehensweise seit der ersten Behandlung von Menschen mit einer Hör-/Sehbeeinträchtigung" verändert? Geben Sie gerne Beispiele an.

3.2 Block B: Barrierefreiheit & Barrieren

Einstiegsfrage: Im Folgenden möchte ich mit Ihnen über das Thema „Barrierefreiheit für Menschen mit einer Hör-/Sehbeeinträchtigung in der Psychotherapie" sprechen. Besonders interessieren mich dabei Ihre Erfahrungen zu diesem Thema. Was verstehen Sie denn allgemein unter dem Begriff „Barrierefreiheit"? Erzählen Sie doch einmal und geben Sie gerne Beispiele an. *(Hinweis für Interviewer: Pause nach der Frage)*

Mögliche Themen/Nachfragen in Block B:

- Eigenes Verständnis von Barrierefreiheit (*Hinweis für Interviewer: Falls nachgefragt wird, was mit „Barrierefreiheit" gemeint ist? Barrieren können Hürden oder Probleme im Alltag sein, die sich durch körperliche oder geistige Beeinträchtigungen ergeben)*
- Wie barrierefrei würden Sie die generelle psychotherapeutische Versorgung für Menschen mit einer Hör-/Sehbeeinträchtigung beurteilen? **(***Hinweis für Interviewer: Bei Verständnisproblemen Beispiel über Schulnoten für die Barrierefreiheit der Psychotherapie stellen, mit 1 = „sehr gut" bis 6 = „ungenügend")*
- Wo sehen Sie in der generellen psychotherapeutischen Versorgung Barrieren für Menschen mit einer Hör-/Sehbeeinträchtigung? Geben Sie gerne Beispiele an. (*Hinweis für Interviewer: Pause nach der Frage*)
    - Barrieren bei der Kontaktaufnahme (Zum Beispiel: Erreichbarkeit per Telefon, E-Mail, etc.)
    - Barrieren in der Kommunikation (Zum Beispiel: non verbal/verbal)
    - Bauliche Barrieren: (Zum Beispiel: Weg zur Therapie, Orientierung im Gebäude, Akustik im Gebäude, etc.)
    - Inhaltliche Barrieren (Zum Beispiel: Einzel-/Gruppenangebote, Diagnostik, Therapie Material, Aufgaben oder Übungen in der Therapie, etc.)
    - Wo liegen für die Patient*innen Ihrer Meinung nach die größten Schwierigkeiten bezogen auf die Barrieren?

  - Warum glauben Sie gibt es diese Barrieren in der Psychotherapie?
- In wie weit decken sich diese generellen Barrieren mit Ihren eigenen Erfahrungen in der psychotherapeutischen Arbeit mit hör-/sehbeeinträchtigten Patient*innen? Geben Sie gerne Beispiele über erlebte Barrieren an. *(Hinweis für Interviewer: Pause nach der Frage)*
  - Barrieren bei der Kontaktaufnahme (Zum Beispiel: Erreichbarkeit per Telefon, E-Mail, etc.)
  - Barrieren in der Kommunikation (Zum Beispiel: non verbal/verbal)
  - Bauliche Barrieren: (Zum Beispiel: Weg zur Therapie, Orientierung im Gebäude, Akustik im Gebäude, etc.)
  - Inhaltliche Barrieren (Zum Beispiel: Einzel-/Gruppenangebote, Diagnostik, Therapie Material, Aufgaben oder Übungen in der Therapie, etc.)
  - Wo liegen für die Patient*innen Ihrer Meinung nach die größten Schwierigkeiten bezogen auf die Barrieren?
  - Warum glauben Sie gibt es diese Barrieren in der Psychotherapie?
- Gab es besondere Herausforderungen für Sie als Psychotherapeut*in in der Arbeit mit hör-/sehbeeinträchtigten Patient*innen? Geben Sie gerne Beispiele an. (*Hinweis für Interviewer: Pause nach der Frage)*
  - Gab es auch Grenzen, an die Sie gestoßen sind? Bitte geben Sie Beispiele an.
- Welchen Umgang mit Menschen mit Behinderung möchten Sie in Ihrer Psychotherapie vermitteln? *(Hinweis für Interviewer: Pause nach der Frage)*
  - Wie reagieren die Patient*innen auf Ihren Umgang mit dem Thema „Behinderung"? Geben Sie gerne Beispiele an.
  - Hat sich Ihr Umgang mit hör-/sehbeeinträchtigten Patient*innen im Laufe der Zeit verändert? Geben Sie gerne Beispiele an.

3.3 Block C: Bewältigungsstrategien & Therapiegestaltung

*Einstiegsfrage***:** Nun haben Sie mir viel über die erlebten Barrieren in Ihrer Arbeit als Psychotherapeut*in berichtet. Bezogen auf diese Barrieren in der Psychotherapie würde mich sehr interessieren, wie Sie und Ihre Patient*innen diese bewältigen. Versuchen Sie sich einmal daran zu erinnern und geben Sie gerne Beispiele an. *(Hinweis für Interviewer: Pause nach der Frage)*

Mögliche Themen/Nachfragen in Block C:

- Was haben Sie als Psychotherapeut*in genau getan, um Barrieren in der Psychotherapie zu überwinden?
- Was haben Ihre Patient*innen getan, um die Barrieren in der Psychotherapie zu überwinden?
- Welche konkreten Hilfen haben Sie bei der Bewältigung von Barrieren in Anspruch genommen? Geben Sie gerne Beispiele an. (*Hinweis für Interviewer: Pause nach der Frage)*
    - Techniken/Strategien in der Psychotherapie (Zum Beispiel: Anpassung der Psychotherapie an die Hör-/Sehbeeinträchtigung der Person)
    - Einsatz von technischen Hilfsmitteln (Zum Beispiel: Sehhilfe/Lupe, Hörgerät, etc.)
    - Wichtige Personen neben Psychotherapeut*in (Zum Beispiel: Gebärdensprachdolmetscher*innen, Assistenzkraft, Mitpatient*innen, Klinik-/Pflegepersonal, Angehörige, Freunde, etc.)
    - Kontakt zu Gemeinschaften/Communities (Zum Beispiel: Verbände, Vereine, Selbsthilfegruppen für hör-/sehbeeinträchtigte Menschen, etc.)
        - Hat Ihr Kontakt zu Gemeinschaften/Communities Ihr Bild von Psychotherapie für Menschen mit einer Hör-/Sehbeeinträchtigung beeinflusst? Wenn ja, wie genau?
- Wie gestalten Sie Ihre Psychotherapie für Menschen mit einer Hör-/Sehbeeinträchtigung möglichst barrierefrei? Geben Sie gerne Beispiele an. *(Hinweis für Interviewer: Pause nach der Frage)*
    - Folgen Sie dabei bestimmten wissenschaftlichen Ansätzen, Artikeln oder Theorien?
    - Gibt es Besonderheiten in der Fallkonzeption bzgl. der Planbarkeit?
    - Was hat bezüglich Ihrer Arbeit mit hör-/sehbeeinträchtigten Patient*innen in der Psychotherapie gut geklappt? Was haben Sie als besonders schöne Erfahrung in Erinnerung behalten?
- Gibt es Ressourcen und Stärken, die Ihnen in der Psychotherapie bei hör-/sehbeeinträchtigten Patient*innen aufgefallen sind? Geben Sie gerne Beispiele an. *(Hinweis für Interviewer: Pause nach der Frage)*
    - Ressourcen/Stärken bei Ihnen als Psychotherapeut*in
    - Ressourcen/Stärken, die durch die Psychotherapie vermittelt wurden

- Können Psychotherapeut*innen womöglich etwas für sich aus der Arbeit mit hör-/sehbeeinträchtigten Patient*innen lernen? Geben Sie gerne Beispiele an.

3.4 Block D: Reflexion

Einstiegsfrage: Ab diesem Punkt neigt sich unser Interview langsam dem Ende zu. Abschließend würde mich noch Ihre Meinung zum Thema „Barrierefreie Psychotherapie für Menschen mit einer Hör-/Sehbeeinträchtigung“ interessieren. Wenn Sie ein abschließendes Resümee über Ihre eigenen Erfahrungen als Psychotherapeut*In für hör-/sehbeeinträchtigte Patient*innen ziehen müssten, wie würde ein solches Resümee lauten? Welche Punkte, die Sie in Ihr Resümee aufnehmen, würden Sie als positiv oder auch negativ bewerten? (*Hinweis für Interviewer: Pause nach der Frage)*

Mögliche Themen/Nachfragen in Block D:

- Wie bewerten Sie die von Ihnen erlebte Barrierefreiheit in der eigenen Psychotherapie? (*Hinweis für Interviewer: Pause nach der Frage)*
  - Erfahrungen mit Dolmetscher*innen/Assistenzkräften bewerten
- Was würden Sie sich für die zukünftige psychotherapeutische Versorgung für Menschen mit einer Hör-/Sehbeeinträchtigung wünschen? Bitte geben Sie Beispiele an. (*Hinweis für Interviewer: Pause nach der Frage)*
  - Wie sähen Ihre konkreten Veränderungsvorschläge aus?
- Was würden Sie jemanden mit einer Hör-/Sehbeeinträchtigung mit auf den Weg geben, der mit dem Gedanken spielt, eine Psychotherapie zu machen?
- Was würden Sie Psychotherapeut*innen raten, die noch nie mit hör-/sehbeeinträchtigten Patient*innen gearbeitet haben und nun vor dieser Aufgabe stehen?
  - Welche Inhalte sind für Psychtotherapeut*innen besonders relevant, wenn Sie noch unerfahren in der Arbeit mit hör-/sehbeeinträchtigten Patient*innen sind?
  - Sehen Sie Schwierigkeiten bei Psychotherapeut*innen, die noch keine Erfahrung in der therapeutischen Arbeit mit hör-/sehbeeinträchtigten Patient*innen gemacht haben?

4. Ausklang & Verabschiedung

Zum Abschluss unseres Interviews würde ich Sie gerne noch fragen:

- Möchten Sie noch etwas ergänzen oder sagen, was bislang noch nicht gesagt oder angesprochen wurde?
- Haben Sie noch Fragen zum Interview oder zum Forschungsprojekt?
- Wie haben Sie sich vor / während / nach dem Interview gefühlt?
- Kontaktmöglichkeiten bei Rückfragen und Interesse am Forschungsprojekt
- Überprüfung der Kontaktadresse für Rücksendung der Antworten zur Kontrolle
- Ort, Datum, Uhrzeit dokumentieren

Vielen Dank für Ihre Zeit. Damit stoppe ich nun die Aufnahme und beende das Interview.

5. Post Scriptum Eindruck

- *Hinweis für Interviewer*: Skizzieren von Auffälligkeiten, die direkt nach dem Interview vorgenommen werden können (z.B. auffälliges Verhalten/auffällige Stimmung während des Interviews)

Interview Guide for Mental Health Professionals (English Translation)

Interview Sections

1. Introduction & Instructions
2. Short Questionnaire
3. Main Section
    3.1 Block A: Providing Mental Health Services for persons with a hearing or vision impairment
    3.2 Block B: Accessibility & Barriers
    3.3 Block C: Accessibility-Improving Strategies & Therapy Expertise
    3.4 Block D: Reflection
4. Conclusion & Closing Remarks
5. Post Scriptum Impressions

1. Introduction & Instructions

First of all, thank you for taking the time and showing interest in answering a few questions today. Before we begin the interview, I would like to briefly tell you a bit about the background of the interview and provide you with some information about how it will proceed. This way, you'll have a clearer idea of what to expect.

Purpose of the Interview

> So, what is this conversation about? As mentioned beforehand, I would like to ask you some in-depth questions about how accessible you experienced psychotherapy as a psychotherapist for clients with a hearing or vision impairment. I'm especially interested in your personal experiences and expertise in this area.

What does this mean for our interview – for my questions and your answers?

> It means that your personal and entirely subjective opinions will be at the center of our conversation. Consequently, there are no right or wrong answers. Everyone has different perspectives. So, please feel free to share whatever comes to mind in response to each question.

Interview Guide

The questions I will ask you are written down in front of me. I may occasionally glance at my notes or jot something down. This is just for my own orientation and should not disturb or unsettle you.

Recording

Since it would be impossible for me to write everything down during the interview, I would like to record our conversation – with your consent, of course. This allows me to give you my full attention during the interview. You may always let me know if there is something you said that you would like to have deleted afterward. This is easier to manage with the recording device I use than stopping the recording during the interview, which would create multiple files. If you wish, I can provide you with a written transcript of our conversation.

Duration

The interview is expected to take approximately 60 to 90 minutes. If you need a break at any point, just let me know. The recording will be paused during any break.

Confidentiality and Anonymity

A few notes on the confidentiality of your statements: In previous interviews, it has worked well for participants to choose a code name to identify themselves. This way, you will be able to recognize your own responses later, while others will not be able to tell who made which statement.

Which code name would you like to choose?

Do you have any questions about the interview so far?

Then I will now start the recording and begin the interview.

2. Short Questionnaire

At the beginning of the interview, I would like to ask you a few general questions.

1. How old are you?

2. What gender do you identify with?
3. What nationality/nationalities do you hold?
4. What is your religious affiliation (if any)?
5. What is your highest level of education? (Please include academic degrees, if applicable)
6. Do you have a hearing or vision impairment yourself?
    - If yes: What is the medical diagnosis of your hearing or vision impairment?
    - To what extent does this diagnosis affect your hearing (in dB) or vision (visual acuity in %)?
    - Do you have a registered degree of disability documented in a disability ID card?
        - If yes: What is your degree of disability?
        - If you have a disability ID card: Do you know which codes are listed in it? (For example: "Gl" for deaf, "Bl" for blind, etc.)
    - Was your hearing or vision impairment congenital or acquired during your life?
        - If acquired: How old were you when it first became noticeable?
    - How has your impairment progressed since it first occurred? (e.g., stable, improved, worsened)
    - Do you have any additional medical diagnoses besides your hearing/vision impairment? If so, which ones?
7. What is your current professional occupation?
    - How long have you been working as a psychotherapist? (in months/years)
    - In which psychotherapeutic approaches or modalities have you been trained? (For example: cognitive behavioral therapy)
    - In what kind of work setting do you practice as a psychotherapist? (For example: private practice, solo or group practice, inpatient therapy in a hospital, partial hospitalization in a day clinic, etc.)
    - Do you exclusively treat clients with private or public health insurance?
8. How many clients with a hearing or vision impairment do you usually have? (In relation to your total number of clients)

- How many persons with a hearing or vision impairment have you treated during your entire career as a psychotherapist?
- How long have you been working with clients who have a hearing or vision impairment? (In months or years)

3. Main Section

3.1 Block A: Providing Mental Health Services for persons with a hearing or vision impairment

Initial question: You have already shared some information about your work as a psychotherapist. I would now like to learn more about your personal experiences as a psychotherapist working with clients who have a hearing or vision impairment. Try to recall how you first came to work with persons who have a hearing or vision impairment. Could you tell me a bit about how that happened for you?

(Note for interviewer: Pause after the initial question)

Possible topics/follow-up questions in Block A:

- *(Note for interviewer: If multiple experiences are present, please talk about whatever comes to mind that you feel like sharing.)*
- What were your first points of contact with the topic of "psychotherapy for persons with a hearing or vision impairment"? Feel free to give examples.
- How did your path toward providing psychotherapy for persons with a hearing or vision impairment unfold?
- Where did you acquire your knowledge about different types of hearing and vision impairments? Feel free to give examples.
- What were your own expectations as a psychotherapist before treating persons with a hearing or vision impairment for the first time? (For example: hopes and/or reservations)
- How has your approach changed since your first treatment of persons with a hearing or vision impairment? Feel free to give examples.

3.2 Block B: Accessibility & Barriers

Initial question: I would now like to talk with you about the topic of "accessibility for persons with a hearing or vision impairment in psychotherapy." I am especially interested in learning about your experiences on this topic. What does the term "accessibility" mean to you in general? Please tell me about it and feel free to give examples.

(Note for interviewer: Pause after the question)

Possible topics/follow-up questions in Block B:

- Personal understanding of accessibility (Note for interviewer: If asked what is meant by "accessibility": Barriers can be obstacles or problems in everyday life that arise from physical or mental impairments)
- How would you assess the general accessibility of psychotherapeutic care for persons with a hearing or vision impairment? (*Note for interviewer: In case of comprehension difficulties, provide an example using a school grading scale for accessibility in psychotherapy, with 1 = "very good" to 6 = "inadequate")*
- Where do you see barriers in general psychotherapeutic care for persons with a hearing or vision impairment? Feel free to give examples. *(Note for interviewer: Pause after the question)*
    - Barriers in contacting a therapist (For example: accessibility by phone, email, etc.)
    - Barriers in communication (For example: non-verbal/verbal)
    - Building-related or environmental barriers (For example: way to the therapy location, navigation within the building, acoustics in the building, etc.)
    - Content-related barriers (For example: individual/group offers, diagnostics, therapy materials, tasks or exercises in therapy, etc.)
    - In your opinion, where do the biggest challenges lie for the clients in relation to these barriers?
    - Why do you think these barriers exist in psychotherapy?
- To what extent do these barriers align with your personal experiences in psychotherapeutic work with persons who have a hearing or vision impairment? Feel free to provide examples of barriers you have encountered. *(Note for interviewer: Pause after the question)*
    - Barriers in contacting a therapist (For example: accessibility by phone, email, etc.)

   - Barriers in communication (For example: non-verbal/verbal)
   - Building-related or environmental barriers (For example: way to the therapy location, navigation within the building, acoustics in the building, etc.)
   - Content-related barriers (For example: individual/group offers, diagnostics, therapy materials, tasks or exercises in therapy, etc.)
   - In your opinion, where do the biggest challenges lie for the clients in relation to these barriers?
   - Why do you think these barriers exist in psychotherapy?
- Have there been particular challenges for you as a psychotherapist in working with persons who have a hearing or vision impairment? Feel free to provide examples. *(Note for interviewer: Pause after the question)*
   - Have there also been limits you have encountered? Please provide examples.
- What kind of way of interacting with persons with disabilities do you aim to foster in your psychotherapy? *(Note for interviewer: Pause after the question)*
   - How do your clients respond to your way of interacting with the topic of "disability"? Feel free to provide examples.
   - Has your way of interacting with clients who have a hearing or vision impairment changed over time? Feel free to provide examples.

3.3 Block C: Accessibility-Improving Strategies & Therapy Expertise

Initial question: You have now told me a lot about the barriers you have experienced in your work as a psychotherapist. With regard to these barriers in psychotherapy, I would be very interested to know how you and your clients manage to overcome these barriers. Try to recall some situations and feel free to give examples.

(Note for interviewer: Pause after the question)

Possible topics/follow-up questions in Block C:

- What exactly have you done as a psychotherapist to overcome barriers in psychotherapy?
- What have your clients done to overcome barriers in psychotherapy?
- What specific forms of support have you used to overcome barriers? Feel free to give examples. *(Note for interviewer: Pause after the question)*

  - Techniques/strategies in psychotherapy (For example: adapting psychotherapy to the person's hearing or vision impairment)
  - Use of assistive devices/technical aids (For example: visual aids/magnifiers, hearing aids, etc.)
  - Important persons besides the psychotherapist (For example: sign language interpreters, assistants, other clients, hospital/care staff, family members, friends, etc.)
  - Contact with communities (For example: associations, clubs, self-help groups for persons with a hearing or vision impairment, etc.)
    - Has your contact with communities influenced your view of psychotherapy for persons with a hearing or vision impairment? If yes, how exactly?
- How do you make your psychotherapy as accessible as possible for persons with a hearing or vision impairment? Feel free to give examples. *(Note for interviewer: Pause after the question)*
  - Do you follow any specific scientific approaches, articles, or theories in this regard?
  - Are there particular aspects in case conceptualization related to planning?
  - What has worked well in your psychotherapy with persons who have a hearing or vision impairment? What have you remembered as a particularly positive experience?
- Are there resources and strengths you have noticed in psychotherapy with persons who have a hearing or vision impairment? Feel free to give examples. *(Note for interviewer: Pause after the question)*
  - Resources/strengths in yourself as a psychotherapist
  - Resources/strengths that were fostered through psychotherapy
- Can psychotherapists perhaps learn something for themselves from working with persons who have a hearing or vision impairment? Feel free to give examples.

3.4 Block D: Reflection

Initial question: From this point on, our interview is slowly coming to an end. To conclude, I would like to hear your opinion on the topic of "accessible psychotherapy for persons with a hearing or vision impairment." If you had to give a final reflection based on your experiences

as a psychotherapist working with persons who have a hearing or vision impairment, what would that reflection be? Which points in your final reflection would you consider as positive, and which as negative?

(Note for interviewer: Pause after the question)

Possible topics/follow-up questions in Block D:

- How do you evaluate the level of accessibility you have experienced in your own psychotherapy? *(Note for interviewer: Pause after the question)*
    - Evaluate your experiences with interpreters/assistants
- What would you wish for the future of psychotherapeutic care for persons with a hearing or vision impairment? Please give examples. *(Note for interviewer: Pause after the question)*
    - What would your concrete suggestions for change look like?
- What would you like to share with someone who has a hearing or vision impairment and is considering starting psychotherapy?
- What advice would you give to psychotherapists who have never worked with persons who have a hearing or vision impairment and are now faced with this task? *(Note for interviewer: Pause after the question)*
    - What content do you think is particularly relevant for psychotherapists who have little or no experience working with persons with a hearing or vision impairment?
    - Do you see any challenges for psychotherapists who have no prior experience in working therapeutically with persons with a hearing or vision impairment?

4. Conclusion & Closing Remarks

At the end of our interview, I would like to ask you a few final questions:

- Would you like to add anything or mention something that has not yet been said or addressed?
- Do you have any questions about the interview or the research project?
- How did you feel before / during / after the interview?
- Contact information for further questions and interest in the research project
- Verification of the contact address for sending the interview transcript for review

- Documentation of place, date, and time

Thank you very much for your time. I will now stop the recording and end the interview.

5. Post Scriptum Impressions

- *Note for interviewer*: Outline any notable observations that can be recorded directly after the interview (e.g., noticeable behavior/noticeable mood during the interview)

Interview Guide for Clients (German Original)

Interviewkapitel

1. Einleitung & Instruktion
2. Kurzfragebogen
3. Hauptteil
    3.1 Block A: Zugang zur Psychotherapie als Person mit einer Hör-/ Sehbeeinträchtigung
    3.2 Block B: Barrierefreiheit & Barrieren
    3.3 Block C: Bewältigungsstrategien
    3.4 Block D: Reflexion
4. Ausklang & Verabschiedung
5. Post Scriptum Eindruck

1. Einleitung & Instruktion

Erst einmal vielen Dank, dass Sie die Zeit und das Interesse aufgebracht haben, mir heute einige Fragen zu beantworten. Bevor wir in das Interview einsteigen, möchte ich Ihnen kurz etwas über den Hintergrund des Interviews erzählen und Ihnen einige Informationen zum Interviewverlauf geben, damit Sie sich ein genaueres Bild davon machen können, was Sie erwartet.

Sinn und Zweck des Interviews

Also: Worum soll es nun in dem folgenden Gespräch gehen? Wie schon vorab erwähnt, möchte ich Ihnen einige vertiefende Fragen dazu stellen, wie barrierefrei Sie Ihre Psychotherapie als Patient*in mit einer Hör-/Sehbeeinträchtigung erlebt haben. Hierbei interessieren mich besonders Ihre eigens gemachten Erlebnisse und Erfahrungen in diesem Bereich.

Was bedeutet das nun konkret für unser Interview – also für meine Fragen und Ihre Antworten?

Es bedeutet, dass während des gesamten Interviews Ihre persönlichen und ganz subjektiven Meinungen Mittelpunkt des Gesprächs sein werden. Folglich gibt es keine

richtigen oder falschen Antworten. Jeder Mensch vertritt schließlich unterschiedliche Meinungen. Von daher können Sie frei und ungezwungen alles berichten, was Ihnen zu der jeweiligen Frage in den Sinn kommt.

Interviewleitfaden

Die Fragen, die ich Ihnen stellen möchte, habe ich für mich schriftlich festgehalten. Ich werde Zwischendurch in meine Unterlagen reinschauen und Notizen machen. Das dient einfach meiner eigenen Orientierung, soll Sie aber nicht stören oder verunsichern.

Aufnahme

Da es mir unmöglich ist, dieses Gespräch vollständig mitzuschreiben, möchte ich es – mit Ihrer Zustimmung versteht sich – aufnehmen. Das hat den Vorteil, dass ich meine volle Aufmerksamkeit auf unser Gespräch lenken kann. Während des Interviews gibt es für Sie immer die Möglichkeit, mir Antworten, die gelöscht werden sollen, mitzuteilen. Das ist bei dem von mir verwendeten Aufnahmegerät hinterher einfacher, als während des Interviews die Aufnahme zu stoppen, da in diesem Falle unterschiedliche Dateien im Gerät erstellt werden. Wenn Sie möchten, lasse ich Ihnen gerne ein verschriftlichtes Exemplar unserer Unterhaltung zukommen.

Dauer

Für das Interview sind ca. 60 – 90 Minuten eingeplant. Sollten Sie während des Interviews eine Pause benötigen, dann geben Sie mir gerne Bescheid. Während dieser Pause würde das Aufnahmegerät gestoppt werden.

Vertraulichkeit und Anonymität

Nun noch einige Hinweise zur Anonymität Ihrer Aussagen: In anderen Interviews hat es sich gut bewährt, dass die einzelnen Teilnehmer*innen zur Kennzeichnung ihrer Person einen Codenamen wählen. So können Sie Ihre Aussagen später erkennen, wohingegen für Dritte nicht ersichtlich ist, welche Person sich hinter der jeweiligen Aussage verbirgt.

Welchen Codenamen möchten Sie für sich wählen?

Haben Sie bis hierin Fragen zum Interview?

Dann beginne ich jetzt mit der Aufnahme und mit dem Interview:

2. Kurzfragebogen

Zu Beginn des Interviews würde ich Ihnen gerne ein paar allgemeine Fragen stellen.

1. Wie alt sind Sie?
2. Welchem Geschlecht fühlen Sie sich zugehörig?
3. Welche Staatsangehörigkeiten besitzen Sie?
4. Zu welcher Konfession/Glaubensrichtung zählen Sie sich?
5. Welcher aktuellen beruflichen Tätigkeit gehen Sie nach?
6. Was ist Ihr höchster Bildungsabschluss? (auch akademische Titel, falls dieser vorhanden ist)
7. Wie lautet die Diagnose Ihrer Hör-/Sehbeeinträchtigung?
    - In wieweit beeinträchtigt diese Diagnose Ihr Hörvermögen in dB /Ihr Sehvermögen (Visus) in Prozent?
    - Haben Sie einen eingetragenen Grad der Behinderung in einem Schwerbehindertenausweis?
        - Wenn Ja: Wie hoch ist Ihr Grad der Behinderung?
        - Wenn ein Schwerbehindertenausweis vorhanden ist: Wissen Sie welche Merkzeichen in Ihrem Schwerbehindertenausweis eingetragen sind? (Zum Beispiel: „Gl“ für gehörlos / „Bl“ für blind, etc.)
    - Ist Ihre Hör-/Sehbeeinträchtigung angeboren oder im Laufe Ihres Lebens aufgetreten?
        - Wenn im Laufe des Lebens aufgetreten: Wie alt waren Sie, als sich Ihre Hör-/Sehbeeinträchtigung zuerst bemerkbar machte?
    - Wie war der Verlauf Ihrer Hör-/Sehbeeinträchtigung nach dem ersten Auftreten? (Zum Beispiel: stabil, verbessert, verschlechtert, etc.)
    - Haben Sie neben Ihrer Hör-/Sehbeeinträchtigung noch weitere eingetragene Diagnosen? Wenn ja, welche?
8. Wann haben Sie in Ihrem Leben zuletzt eine Psychotherapie in Anspruch genommen? (vor Monaten/Jahren)
    - In welchem Rahmen findet/fand Ihre Psychotherapie statt? (Ambulant/stationär/ teilstationär, etc.)

- Über welchen Zeitraum lief Ihre Psychotherapie? (Jahre/Monate/Wochen und Frequenz/Abstände zwischen den Sitzungen)
- Befinden Sie sich aktuell noch in psychotherapeutischer Behandlung?
- Wissen Sie nach welcher Therapieform Ihre Psychotherapie ausgelegt war? (Zum Beispiel: Verhaltenstherapie/kognitive Verhaltenstherapie/Psychoanalyse/psychodynamische Psychotherapie/systemische Therapie, etc.)
- Handelt es sich bei Ihrer Psychotherapie um ein Angebot für privat oder gesetzlich krankenversicherte Patient*innen?
- Aufgrund welcher Diagnose befinden/befanden Sie sich in psychotherapeutischer Behandlung?

9. Hatten Sie vor Ihrer letzten psychotherapeutischen Behandlung bereits Erfahrung mit Psychotherapie als Patient*in?
    - *(Hinweis für Interviewer**:** Wenn ja, Sprung zu Frage 8. um Mehrfachbehandlung zu skizzieren, falls vorhanden)*

3. Hauptteil

3.1 Block A: Zugang zur Psychotherapie als Person mit einer Hör-/Sehbeeinträchtigung

*Einstiegsfrage***:** Nun haben Sie mir bereits von einigen Informationen über Ihre Psychotherapie berichtet. Gerne würde ich mehr über Ihre persönlichen Erfahrungen als Patient*in in der Psychotherapie sprechen. Versuchen Sie sich einmal daran zu erinnern, wie Sie zu Ihrer psychotherapeutischen Behandlung gekommen sind. Erzählen Sie doch einmal, wie das bei Ihnen war. *(Hinweis für Interviewer: Pause nach der Einstiegsfrage)*

Mögliche Themen/Nachfragen in Block A:

- *(Hinweis für Interviewer: Wenn mehrere Therapieerfahrungen vorhanden sind, Sprechen Sie bitte über das, was Ihnen gerade in den Sinn kommt, worüber Sie sprechen möchten)*
- Wie haben Sie Ihre(n) Psychotherapeut*in gefunden? (*Hinweis für Interviewer*: Pause nach der Frage)
    - Hatten Sie Schwierigkeiten bei der Suche nach einem Psychotherapieplatz/Psychotherapeut*in? Wenn ja, welche?

  - Haben Sie Wartezeit in Kauf nehmen müssen? Wenn ja, wieso kam diese zustande?
  - Wissen Sie etwas über den Ablauf der Antragstellung für die Psychotherapie bei Ihrer Krankenkasse?
- Wie waren Ihre eigenen Erwartungen an die Psychotherapie vor der Behandlung? (Zum Beispiel: Wünsche und/oder Vorbehalte)
- Wie lief Ihr Weg als Patient*in in der Psychotherapie ab? (Zum Beispiel: Verlauf der Therapie: Hat sich durch die Therapie etwas bei Ihnen verändert?) *(Hinweis für Interviewer: Pause nach der Frage)*
  - Haben Sie die Psychotherapie abgeschlossen/abgebrochen/läuft Sie noch?
  - Wenn abgebrochen: Wieso haben Sie Ihre Psychotherapie abgebrochen/nicht beendet?
- Können für andere Menschen mit einer Hör-/Sehbeeinträchtigung Probleme entstehen, wenn es um den Zugang zu einer Psychotherapie geht? (Zum Beispiel: das Suchen und Finden eines Psychotherapieplatzes)

3.2 Block B: Barrierefreiheit & Barrieren

Einstiegsfrage: Im Folgenden möchte ich mit Ihnen über das Thema „Barrierefreiheit für Menschen mit einer Hör-/Sehbeeinträchtigung in der Psychotherapie" sprechen. Besonders interessieren mich dabei Ihre Erfahrungen zu diesem Thema. Was verstehen Sie denn allgemein unter dem Begriff „Barrierefreiheit"? Erzählen Sie doch einmal und geben Sie gerne Beispiele an. (*Hinweis für Interviewer: Pause nach der Frage)*

Mögliche Themen/Nachfragen in Block B:

- Eigenes Verständnis von Barrierefreiheit (*Hinweis für Interviewer**:** Falls nachgefragt wird, was mit „Barrierefreiheit" gemeint ist? Barrieren können Hürden oder Probleme im Alltag sein, die sich durch körperliche oder geistige Beeinträchtigungen ergeben*)
- Wie barrierefrei war Ihre Psychotherapie für Sie? **(***Hinweis an Interviewer**:** Bei Verständnisproblemen Beispiel über Schulnoten für die Barrierefreiheit der Psychotherapie stellen, mit 1 = „sehr gut" bis 6 = „ungenügend")*
- Welche Barrieren haben Sie in Ihrer Psychotherapie selbst erlebt? Geben Sie gerne Beispiele an. (*Hinweis für Interviewer: Pause nach Frage)*

  - Barrieren bei der Kontaktaufnahme (Zum Beispiel: Erreichbarkeit per Telefon, E-Mail, etc.)
  - Barrieren in der Kommunikation (Zum Beispiel: non verbal/verbal)
  - Bauliche Barrieren: (Zum Beispiel: Weg zur Therapie, Orientierung im Gebäude, Akustik im Gebäude, etc.)
  - Inhaltliche Barrieren: (Zum Beispiel: Einzel-/Gruppenangebote, Diagnostik, Therapie Material, Aufgaben oder Übungen in der Therapie, etc.)
  - Was hat Ihnen konkret nicht gutgetan in der Psychotherapie?
  - Warum glauben Sie gibt es diese Barrieren in der Psychotherapie für Menschen mit einer Hör-/Sehbeeinträchtigung?
- Gab es besondere Herausforderungen für Sie als Patient*in mit einer Hör-/Sehbeeinträchtigung in der Psychotherapie? Geben Sie gerne Beispiele an. *(Hinweis für Interviewer: Pause nach der Frage)*
  - Gab es auch Grenzen, an die Sie gestoßen sind? Bitte geben Sie Beispiele an.
- Wie ist Ihr(e) Psychotherapeut*in mit Ihrer Hör-/Sehbeeinträchtigung umgegangen, als Er/Sie davon erfahren hat? Geben Sie gerne Beispiele an. *(Hinweis für Interviewer: Pause nach Frage)*
  - Wie haben Sie auf Ihre(n) Psychotherapeut*in reagiert?
  - Hat sich der Umgang mit Ihrer Beeinträchtigung im Laufe der Psychotherapie verändert? Geben Sie gerne Beispiele an.
- Wie barrierefrei schätzen Sie die allgemeine psychotherapeutische Versorgung für Menschen mit einer Hör-/Sehbeeinträchtigung ein? *(Hinweis für Interviewer: Beispiel über Schulnoten)*

3.3 Block C: Bewältigungsstrategien

Einstiegsfrage: Nun haben Sie mir viel über die erlebten Barrieren in Ihrer Psychotherapie berichtet. Bezogen auf diese Barrieren in der Psychotherapie würde mich sehr interessieren, wie Sie und Ihr(e) Psychotherapeut*in diese bewältigt haben. Versuchen Sie sich einmal daran zu erinnern und geben Sie gerne Beispiele an. (*Hinweis für Interviewer: Pause nach der Frage)*

Mögliche Themen/Nachfragen in Block C:

- Was haben Sie als Patient*in genau getan, um Barrieren in der Psychotherapie zu überwinden?

- Was hat Ihr(e) Psychotherapeut*in getan, um die Barrieren in der Psychotherapie zu überwinden?
- Welche konkreten Hilfen haben Sie bei der Bewältigung von Barrieren in Anspruch genommen? Geben Sie gerne Beispiele an. (*Hinweis für Interviewer: Pause nach der Frage)*
    - Techniken/Strategien in der Psychotherapie (Zum Beispiel: Anpassung der Psychotherapie an die Hör-/Sehbeeinträchtigung der Person)
    - Einsatz von technischen Hilfsmitteln (Zum Beispiel: Sehhilfe/Lupe, Hörgerät, etc.)
    - Wichtige Personen neben Psychotherapeut*in (Zum Beispiel: Gebärdensprachdolmetscher*innen, Assistenzkraft, Mitpatient*innen, Klinik-/Pflegepersonal, Angehörige, Freunde, etc.)
    - Kontakt zu Gemeinschaften/Communities (Zum Beispiel: Verbände, Vereine, Selbsthilfegruppen für hör-/sehbeeinträchtigte Menschen, etc.)
        - Hat Ihr Kontakt zu Gemeinschaften/Communities Ihr Bild von Psychotherapie beeinflusst? Wenn ja, wie genau?
- Was hat bezüglich Ihrer Hör-/Sehbeeinträchtigung in der Psychotherapie gut geklappt? Was haben Sie als besonders schöne Erfahrung in Erinnerung behalten?
- Gibt es Ressourcen und Stärken (positiven Eigenschaften), die Ihnen in der Psychotherapie bei sich selbst aufgefallen sind? Geben Sie gerne Beispiele an. (*Hinweis für Interviewer: Pause nach der Frage)*
    - Ressourcen/Stärken des/der Psychotherapeut*in
    - Ressourcen/Stärken, die durch die Psychotherapie vermittelt wurden
- Können Psychotherapeut*innen womöglich etwas aus der Arbeit mit hör-/sehbeeinträchtigten Menschen lernen? Geben Sie gerne Beispiele an.

3.4 Block D: Reflexion

Einstiegsfrage: Ab diesem Punkt neigt sich unser Interview langsam dem Ende zu. Abschließend würde mich noch Ihre Meinung zum Thema „Barrierefreie Psychotherapie für Menschen mit einer Hör-/Sehbeeinträchtigung“ interessieren. Wenn Sie ein abschließendes Resümee über Ihre eigenen Erfahrungen als hör-/sehbeeinträchtigte Patient*innen ziehen müssten, wie würde ein solches Resümee lauten? (*Hinweis für den Interviewer: Falls Verständnisfragen aufkommen sollten: rückblickend eine Bilanz ziehen oder eine*

Zusammenfassung abgeben) Welche Punkte, die Sie in Ihr Resümee aufnehmen, würden Sie als positiv oder auch negativ bewerten? (*Hinweis für Interviewer: Pause nach der Frage)*

Mögliche Themen/Nachfragen in Block D:

- Wie bewerten Sie die von Ihnen erlebte Barrierefreiheit in der eigenen Psychotherapie? (*Hinweis für Interviewer: Pause nach der Frage)*
    - Erfahrung mit Dolmetscher*innen/Assistenzkräften bewerten
- Würden Sie sich noch einmal für eine Psychotherapie entscheiden, wenn Sie in eine ähnliche Situation geraten sollten?
    - Würden Sie etwas anders machen als bei Ihrer letzten Psychotherapie?
- Was würden Sie sich für die zukünftige psychotherapeutischen Versorgung für Menschen mit einer Hör-/Sehbeeinträchtigung wünschen? Bitte geben Sie Beispiele an. (*Hinweis für Interviewer**:** Pause nach der Frage)*
    - Wie sähen Ihre konkreten Veränderungsvorschläge aus?
- Was würden Sie einer anderen Person mit einer Hör-/Sehbeeinträchtigung mit auf den Weg geben, der/die mit dem Gedanken spielt, eine Psychotherapie zu machen?
- Was würden Sie Psychotherapeut*innen raten, die noch nie mit hör-/sehbeeinträchtigten Patient*innen gearbeitet haben und nun vor dieser Aufgabe stehen? (*Hinweis für Interviewer**:** Pause nach der Frage)*
    - Welche Inhalte sind für Psychtotherapeut*innen besonders relevant, wenn Sie noch unerfahren in der Arbeit mit hör-/sehbeeinträchtigten Patient*innen sind?
    - Sehen Sie Schwierigkeiten bei Psychotherapeut*innen, die noch keine Erfahrung in der therapeutischen Arbeit mit hör-/sehbeeinträchtigten Patient*innen gemacht haben?

4. Ausklang & Verabschiedung

Zum Abschluss unseres Interviews würde ich Sie gerne noch fragen:

- Möchten Sie noch etwas ergänzen oder sagen, was bislang noch nicht gesagt oder angesprochen wurde?
- Haben Sie noch Fragen zum Interview oder zum Forschungsprojekt?
- Wie haben Sie sich vor / während / nach dem Interview gefühlt?
- Kontaktmöglichkeiten bei psychischen Problemen: Falls Sie nach unserem Interview merken sollten, dass Sie mit einem Psychotherapeuten in Kontakt treten möchten, um

über Probleme zu sprechen, die mit dem heutigen Interview in Verbindung stehen, können Sie sich für ein Gespräch an einen Psychotherapeuten wenden, der als psychologischer Psychotherapeut (in kognitiver Verhaltenstherapie) im Zentrum für psychische Gesundheit und Psychotherapie (ZPP) der Universität Witten/Herdecke tätig ist.

- Kontaktmöglichkeiten bei Rückfragen und Interesse am Forschungsprojekt
- Überprüfung der Kontaktadresse für Rücksendung der Antworten zur Kontrolle
- Ort, Datum, Uhrzeit dokumentieren

Vielen Dank für Ihre Zeit. Damit stoppe ich nun die Aufnahme und beende das Interview.

5. Post Scriptum Eindruck

- *Hinweis für Interviewer:* Skizzieren von Auffälligkeiten, die direkt nach dem Interview vorgenommen werden können (z.B. auffälliges Verhalten / auffällige Stimmung während des Interviews)

Interview Guide for Clients (English Translation)

Interview Sections

1. Introduction & Instructions
2. Short Questionnaire
3. Main Section
    - 3.1 Block A: Accessing Psychotherapy for persons with a hearing or vision impairment
    - 3.2 Block B: Accessibility & Barriers
    - 3.3 Block C: Accessibility-Improving Strategies
    - 3.4 Block D: Reflection
4. Conclusion & Closing Remarks
5. Post Scriptum Impressions

1. Introduction & Instructions

First of all, thank you for taking the time and showing interest in answering a few questions today. Before we begin the interview, I would like to briefly tell you a bit about the background of the interview and provide you with some information about how it will proceed. This way, you'll have a clearer idea of what to expect.

Purpose of the Interview

> So, what is this conversation about? As mentioned beforehand, I would like to ask you some in-depth questions about how accessible you experienced your psychotherapy as a client with a hearing or vision impairment. I'm especially interested in your personal experiences in this area.

What does this mean for our interview – for my questions and your answers?

> It means that your personal and entirely subjective opinions will be at the center of our conversation. Consequently, there are no right or wrong answers. Everyone has different perspectives. So, please feel free to share whatever comes to mind in response to each question.

Interview Guide

The questions I will ask you are written down in front of me. I may occasionally glance at my notes or jot something down. This is just for my own orientation and should not disturb or unsettle you.

Recording

Since it would be impossible for me to write everything down during the interview, I would like to record our conversation—with your consent, of course. This allows me to give you my full attention during the interview. You may always let me know if there is something you said that you would like to have deleted afterward. This is easier to manage with the recording device I use than stopping the recording during the interview, which would create multiple files. If you wish, I can provide you with a written transcript of our conversation.

Duration

The interview is expected to take approximately 60 to 90 minutes. If you need a break at any point, just let me know. The recording will be paused during any break.

Confidentiality and Anonymity

A few notes on the confidentiality of your statements: In previous interviews, it has worked well for participants to choose a code name to identify themselves. This way, you will be able to recognize your own responses later, while others will not be able to tell who made which statement.

Which code name would you like to choose?

Do you have any questions about the interview so far?

Then I will now start the recording and begin the interview.

2. Short Questionnaire

At the beginning of the interview, I would like to ask you a few general questions.

1. How old are you?

2. What gender do you identify with?
3. What nationality/nationalities do you hold?
4. What is your religious affiliation (if any)?
5. What is your current occupation?
6. What is your highest level of education? (Please include academic degrees, if applicable)
7. What is the diagnosis of your hearing or vision impairment?
    - To what extent does this diagnosis affect your hearing (in dB) or vision (visual acuity in %)?
    - Do you have a registered degree of disability documented in a disability ID card?
        - If yes: What is your degree of disability?
        - If you have a disability ID card: Do you know which codes are listed in it? (For example: "Gl" for deaf, "Bl" for blind, etc.)
    - Was your hearing or vision impairment congenital or acquired during your life?
        - If acquired: How old were you when it first became noticeable?
    - How has your impairment progressed since it first occurred? (e.g., stable, improved, worsened)
    - Do you have any additional medical diagnoses besides your hearing/vision impairment? If so, which ones?
8. When was the last time in your life you received psychotherapy? (e.g., months/years ago)
    - In what setting did your psychotherapy take place? (e.g., outpatient, inpatient, day clinic, etc.)
    - Over what period did your psychotherapy take place? (e.g., years/months/weeks, and the frequency/intervals between sessions)
    - Are you currently undergoing psychotherapy?
    - Do you know what type of therapy was used in your treatment? (e.g., cognitive behavioral therapy, psychoanalysis, psychodynamic therapy, systemic therapy, etc.)
    - Was your psychotherapy covered by public or private health insurance?
    - What was the diagnosis that led to your psychotherapy?

9. Did you have any prior experience with psychotherapy as a client before your most recent treatment?
    - *(Note for interviewer: If yes, skip back to question 8 to capture multiple therapy experiences, if applicable)*

3. Main Section

3.1 Block A: Accessing Psychotherapy for persons with a hearing or vision impairment

Initial question: You have already shared some information about your psychotherapy. I would now like to learn more about your personal experiences as a client in psychotherapy. Try to recall how you came to receive psychotherapeutic treatment. Could you tell me a bit about how that happened for you?

(Note for interviewer: Pause after the initial question)

Possible topics/follow-up questions in Block A:

- *(Note for interviewer: If multiple therapy experiences are present, please talk about whatever comes to mind that you feel like sharing.)*
- How did you find your psychotherapist? *(Note for interviewer: Pause after the question)*
    - Did you have any difficulties in finding a place for psychotherapy or a psychotherapist? If so, what kind of difficulties?
    - Did you have to accept a waiting period? If so, what was the reason for this?
    - Are you familiar with the process of applying for psychotherapy coverage with your health insurance?
- What were your own expectations of psychotherapy before starting treatment? (For example: hopes and/or reservations)
- How did your path as a client in psychotherapy unfold? (For example: course of therapy – has anything changed for you through therapy?) *(Note for interviewer: Pause after the question)*
    - Did you complete your psychotherapy, discontinue it, or is it still ongoing?
    - If you discontinued: Why did you end or not complete your psychotherapy?

- Can you imagine that other persons with a hearing or vision impairment might face challenges when it comes to accessing psychotherapy? (For example: searching for and finding a psychotherapist)

3.2 Block B: Accessibility & Barriers

Initial question: I would now like to talk with you about the topic of "accessibility for persons with a hearing or vision impairment in psychotherapy." I am especially interested in learning about your experiences on this topic. What does the term "accessibility" mean to you in general? Please tell me about it and feel free to give examples.

(Note for interviewer: Pause after the question)

Possible topics/follow-up questions in Block B:

- Personal understanding of accessibility (Note for interviewer: If asked what is meant by "accessibility": Barriers can be obstacles or problems in everyday life that arise from physical or mental impairments)
- How accessible was your psychotherapy for you? (*Note for interviewer: In case of comprehension difficulties, provide an example using a school grading scale for accessibility in psychotherapy, with 1 = "very good" to 6 = "inadequate")*
- What barriers did you experience in your psychotherapy? Feel free to give examples. (*Note for interviewer: Pause after the question*)
    - Barriers in contacting a therapist (For example: accessibility by phone, email, etc.)
    - Barriers in communication (For example: non-verbal/verbal)
    - Building-related or environmental barriers (For example: way to the therapy location, navigation within the building, acoustics in the building, etc.)
    - Content-related barriers (For example: individual/group offers, diagnostics, therapy materials, tasks or exercises in therapy, etc.)
    - What specifically did not feel helpful or beneficial to you in psychotherapy?
    - Why do you think these barriers exist in psychotherapy?
- Have there been particular challenges for you as a client with a hearing or vision impairment in psychotherapy? Feel free to give examples. *(Note for interviewer: Pause after the question)*
    - Have there also been limits you have encountered? Please give examples.

- How did your psychotherapist respond when they learned about your hearing or vision impairment? Feel free to give examples. *(Note for interviewer: Pause after the question)*
    - How did you respond to your psychotherapist?
    - Has the way your hearing or vision impairment was addressed changed over the course of psychotherapy? Feel free to give examples.
- How would you assess the general accessibility of psychotherapeutic care for persons with a hearing or vision impairment? *(Note for interviewer: Example with school grading system: 1 = "very good" to 6 = "inadequate")*

3.3 Block C: Accessibility-Improving Strategies

Initial question: You have now told me a lot about the barriers you have experienced in your psychotherapy. With regard to these barriers in psychotherapy, I would be very interested to know how you and your psychotherapist manage to overcome these barriers. Try to recall some situations and feel free to give examples.

(Note for interviewer: Pause after the question)

Possible topics/follow-up questions in Block C:

- What exactly have you done as a client to overcome barriers in psychotherapy?
- What has your psychotherapist done to overcome barriers in psychotherapy?
- What specific forms of support have you used to overcome barriers? Feel free to give examples. *(Note for interviewer: Pause after the question)*
    - Techniques/strategies in psychotherapy (For example: adapting psychotherapy to the hearing or vision impairment)
    - Use of assistive devices/technical aids (For example: visual aids/magnifiers, hearing aids, etc.)
    - Important persons besides the psychotherapist (For example: sign language interpreters, assistants, other clients, hospital/care staff, family members, friends, etc.)
    - Contact with communities (For example: associations, clubs, self-help groups for persons with a hearing or vision impairment, etc.)
        - Has your contact with communities influenced your view of psychotherapy? If yes, how exactly?

- *What do you remember as a particularly positive experience?*
- Are there resources and strengths (positive qualities) you have noticed in yourself during psychotherapy? Feel free to give examples. *(Note for interviewer: Pause after the question)*
    - Resources/strengths of the psychotherapist
    - Resources/strengths that were fostered through psychotherapy
- Can psychotherapists perhaps learn something for themselves from working with persons who have a hearing or vision impairment? Feel free to give examples.

3.4 Block D: Reflection

Initial question: From this point on, our interview is slowly coming to an end. To conclude, I would like to hear your opinion on the topic of "accessible psychotherapy for persons with a hearing or vision impairment." If you had to give a final reflection based on your own experiences as a client with a hearing or vision impairment, what would that reflection be? *(Note for interviewer: In case of comprehension difficulties, explain that a final reflection means drawing a conclusion or giving a summary based on past experiences.)* Which points in your final reflection would you consider as positive, and which as negative?

(Note for interviewer: Pause after the question)

Possible topics/follow-up questions in Block D:

- How would you evaluate the level of accessibility you experienced in your own psychotherapy? *(Note for interviewer: Pause after the question)*
    - Evaluate your experiences with interpreters/assistants
- Would you choose to start psychotherapy again if you were in a similar situation?
    - Would you do anything differently compared to your last psychotherapy?
- What would you wish for the future of psychotherapeutic care for persons with a hearing or vision impairment? Please give examples. *(Note for interviewer: Pause after the question)*
    - What would your concrete suggestions for change look like?

- What would you like to share with another person who has a hearing or vision impairment and is considering starting psychotherapy?
- What advice would you give to psychotherapists who have never worked with clients who have a hearing or vision impairment and are now faced with this task? *(Note for interviewer: Pause after the question)*
    - What content do you think is particularly relevant for psychotherapists who have little or no experience working with persons with a hearing or vision impairment?
    - Do you see any challenges for psychotherapists who have no prior experience in working therapeutically with persons with a hearing or vision impairment?

4. Conclusion & Closing Remarks

At the end of our interview, I would like to ask you a few final questions:

- Would you like to add anything or mention something that has not yet been said or addressed?
- Do you have any questions about the interview or the research project?
- How did you feel before / during / after the interview?
- Contact information in case of psychological difficulties: If you notice after our interview that you would like to get in touch with a psychotherapist to discuss issues related to today's interview, you can contact a psychotherapist who is a licensed psychotherapist (in cognitive behavioral therapy) at the Center for Mental Health and Psychotherapy (ZPP) at Witten/Herdecke University.
- Contact information for further questions and interest in the research project
- Verification of the contact address for sending the interview transcript for review
- Documentation of place, date, and time

Thank you very much for your time. I will now stop the recording and end the interview.

5. Post Scriptum Impressions

- *Note for interviewer*: Outline any notable observations that can be recorded directly after the interview (e.g., noticeable behavior/noticeable mood during the interview)
